# The Modern Numerical and Experimental Methods for the Sound Absorbing Characteristics of Dissipative Sound Absorbing Materials: A Review

**DOI:** 10.3390/ma18235353

**Published:** 2025-11-27

**Authors:** Ruijun Liu, Zhicheng Zhang, Xu Zheng

**Affiliations:** College of Energy Engineering, Zhejiang University, Hangzhou 310027, China; beefman@zju.edu.cn (R.L.); zzc666@zju.edu.cn (Z.Z.)

**Keywords:** dissipative sound-absorbing materials, sound absorption coefficient, numerical methods, experimental methods, machine learning, flow resistivity

## Abstract

This review provides a comprehensive overview of modern experimental and numerical methods for characterizing the sound absorbing properties of dissipative sound-absorbing materials. Experimentally, we summarize both in situ techniques (e.g., pulse reflection, two-microphone, p-u probe, and spatial Fourier transform method) and laboratory methods (e.g., impedance tube, transfer function, and reverberation room methods), discussing their principles and applications. For the numerical methods, we detail the development and refinement of empirical models (e.g., Delany–Bazley, Miki, Komatsu), theoretical models (e.g., Johnson–Champoux–Allard), and computer numerical methods, along with methods for obtaining flow resistivity, including empirical formulas, experimental measurements. Furthermore, we review recent advances in machine learning approaches (e.g., generalized regression neural networks, radial basis function neural networks, and artificial neural networks) for predicting the sound absorption coefficient. This work aims to serve as a methodological reference for the research, development, and performance evaluation of dissipative sound-absorbing materials.

## 1. Introduction

Sound-absorbing materials could be classified as reactive absorbers, dissipative absorbers and composite impedance absorbers [1,2,3]. A reactive absorber consists of pipes and chambers with alterations at the interface, resembling an acoustic filter, which provides attenuation as sound passes through the modified sections of the pipe. The advantages of reactive absorbers lie in their ability to absorb low-frequency noise. However, their drawbacks are also very apparent, such as their narrow sound absorption bandwidth and poor machinability. For dissipative sound-absorbing materials, the typical types include foams, fibers, and other materials with a high internal porosity. These pores are uniformly distributed across the surface and interior of the structure, allowing dissipative sound-absorbing materials to exhibit good sound absorption performance across a wide frequency range, particularly in the high-frequency range, while their sound absorption performance is relatively weaker in the low-frequency range. Due to the low production cost of dissipative sound-absorbing materials, their application scope is gradually expanding. The composite impedance absorber combines the features of the previous two types, achieving a superior sound-absorbing effect, albeit with a larger size. In the following sections of this paper, only the dissipative sound-absorbing materials will be introduced and discussed in detail.

**Figure 1:**
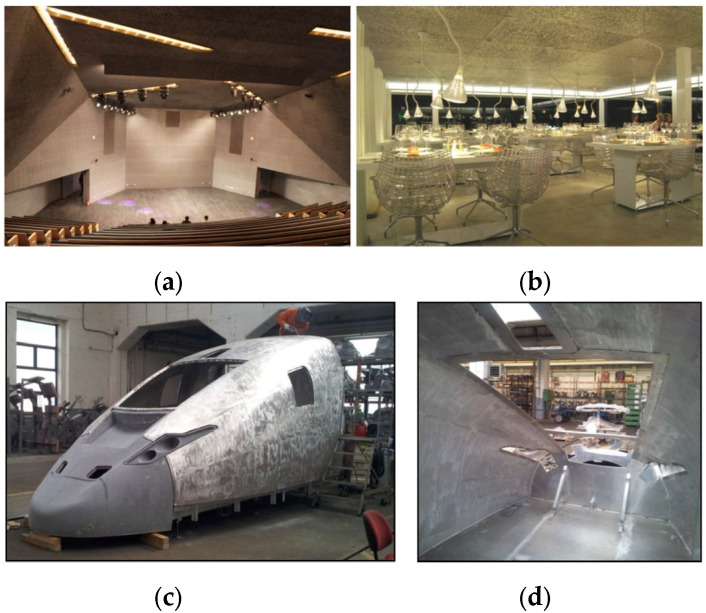
Image of dissipative sound-absorbing materials used in (**a**) audience hall, (**b**) restaurant covered by foams, (**c**) and high-speed train made of welded aluminum alloy foam sandwich [12]. (**d**) Interior view of the high-speed train (reproduced with permission from MDPI).

Dissipative sound-absorbing materials have been widely used in many fields such as automotive, architecture, and aviation, as shown in Figure 1. In the automotive industry, the main application of dissipative sound-absorbing materials is to reduce in-vehicle noise and enhance driving comfort and quietness [4,5,6,7]. These materials are typically used in various parts of the car cabin, such as the roof, door panels, floor, and instrument panel. They can reduce echo inside the vehicle and improve the quality of the audio system. In the aviation field, dissipative sound-absorbing materials used in engine acoustic enclosures and engine compartments help reduce the transmission of engine noise. The low-frequency noise generated by aircraft engines is suppressed by the dissipative sound-absorbing materials in the acoustic enclosures, intake systems, and exhaust systems. The internal cabin noise of an aircraft mainly comes from the engine, airflow, and mechanical vibrations. By using dissipative sound-absorbing materials in the cabin floor, ceiling, window frames, and seats, the impact of these noise sources can be effectively reduced [8,9]. In the construction industry, dissipative sound-absorbing materials are widely used to control noise pollution inside and outside buildings, as shown in Figure 1a,b. Dissipative sound-absorbing materials such as gypsum boards, soundproof cotton, and sound-absorbing foam can effectively reduce noise transmission through walls, ceilings, and floors [10,11,12].

The sound absorption coefficient (SAC) is a significant parameter with which to evaluate the sound energy absorption capability of a structure, and it is usually expressed by *α*. SAC is defined as the ratio of absorbed sound energy to the total incident sound energy. Its expression formula is as follows:

(1) $$E_{i}=E_{r}+E_{t}+E_{\alpha }$$

(2) $$\alpha =\frac{E_{\alpha }}{E_{i}}=\frac{E_{i}-E_{r}-E_{t}}{E_{i}}$$

where $E_{i}$ denotes the total incident sound energy (J), $E_{\alpha }$ denotes the absorbed sound energy (J), $E_{r}$ is the reflected sound energy (J) and $E_{t}$ is the transmitted absorbed energy (J), *α* is the sound absorbing coefficient.

It is important to note that the measured SAC depends strongly on the sound field incidence condition. To avoid ambiguity, we adopt the following subscript notation throughout this review:

$\alpha_{n}$: The normal incidence sound absorption coefficient, typically measured in an impedance tube.

$\alpha_{r}$: The random incidence sound absorption coefficient, measured in a reverberation room.

$\alpha_{i}$: The in situ sound absorption coefficient, measured under real-world conditions, which may involve complex and oblique sound fields.

$\alpha_{a}\left(\theta \right)$: The angle-dependent sound absorption coefficient for a specific incidence angle θ.

The SAC is a key indicator for evaluating the performance of sound-absorbing materials. Its calculation and measurement hold significant importance in the process of material research and development. With advancements in theory and technology, the measurement methods for SAC have undergone long-term evolution.

The experimental testing technology for SAC has developed systematically. In situ measurement methods can be used to evaluate the performance of materials in actual service environments, specifically including the pulse reflection method, two-microphone method, p-u vector sensor method, and spatial Fourier transform method. In contrast, laboratory measurement methods are more widely used in the material research and development stage due to their excellent repeatability and controllability. Examples include the impedance tube method, transfer function method, and p-u vector sensor method (conducted in impedance tubes), as well as the reverberation room method (conducted in reverberation rooms).

In 1970, Delany and Bazley [13] proposed the Delany–Bazley (DB) empirical model based on extensive experimental data, which predicted SAC using the flow resistivity of materials. In 1990, Miki [14,15] optimized the DB model and put forward the Miki empirical model, significantly enhancing the model’s applicability in the low-frequency range. In 2008, Komatsu [16] further improved the aforementioned models, making their prediction of SAC for fibrous sound-absorbing materials more accurate under conditions of extreme density and frequency. In 1991, Champoux and Allard [17], building on the dynamic tortuosity theory proposed by Johnson et al. in 1987 [18], introduced the frequency dependence of dynamic bulk modulus and established the Johnson–Champoux–Allard (JCA) model. This model uniformly describes the viscous and thermal effects of sound propagation in porous media, providing a solid theoretical foundation for the design and optimization of acoustic materials. Among the aforementioned models, flow resistivity is an indispensable input parameter. Currently, there are various methods to obtain flow resistivity, including the empirical Bies and Hansen model [19], experimental measurements that comply with international standards, and numerical simulation-based methods.

In recent years, with the rapid development of machine learning technology, an increasing number of studies have attempted to use data-driven methods to predict the sound absorption performance of materials. These methods train models using large amounts of data and offer advantages of high efficiency and convenience in practical applications. Their prediction accuracy mainly depends on the selection of model structure and the quality and scale of training data. Currently, the methods reported in research include the Generalized Regression Neural Network (GRNN), Radial Basis Function Neural Network (RBFNN), and Artificial Neural Network (ANN), all of which show promising application prospects.

Despite the extensive literature on the acoustic properties of materials and methods for predicting the sound absorption coefficient (SAC), comprehensive review articles in this field remain scarce. Existing reviews often focus on a single aspect, such as comparing a limited number of experimental techniques [20,21], analyzing several empirical models [22,23], or examining the acoustic performance of a specific material type [24]. However, a systematic and integrated methodological framework, which bridges standardized experimental methods, numerical models, and the emerging data-driven paradigm of machine learning (ML), is notably absent. While previous reviews have provided valuable insights within their respective domains, a holistic and comparative analysis that bridges these disparate methodological silos is lacking. This gap poses challenges for researchers and engineers, especially those new to the field or working on innovative materials, in navigating the vast methodological landscape and selecting the optimal combination of techniques for their specific needs.

Therefore, this review seeks to address this critical gap. It aims to provide a unified and critical overview of the modern methodological landscape for determining the SAC of dissipative sound-absorbing materials. This review sets out with the following primary objectives:

1. To systematically catalog and critically evaluate the full spectrum of modern methods for characterizing SAC, encompassing in situ and laboratory experimental techniques, empirical, theoretical, and computational numerical models, and the emerging paradigm of machine learning.
2. To elucidate the interconnections, advantages, and limitations of each method, thereby constructing a coherent methodological framework.
3. To provide a comparative analysis that guides method selection based on accuracy, complexity, cost, and application context.

By integrating these traditionally separate domains into a single, comprehensive narrative, this review pushes the current understanding forward. It moves beyond a simple cataloging of methods to offer a decision-making framework for acoustic material characterization. This integrated perspective is particularly timely, given the growing convergence of physics-based modeling and data-driven approaches in materials science. Consequently, this work stands out by serving not only as a reference for the current state-of-the-art but also as a roadmap for the future development and application of hybrid methodologies in acoustic materials research.

## 2. Experimental Methods for SAC

### 2.1. In Situ Measurement Methods

The in situ measurement method is suitable for evaluating the acoustic performance of installed materials and is closer to the actual application environment. However, it is limited by background noise, sound field conditions, and other factors. Common methods include the impulse reflection method, two-microphone method, p-u probe method, and sound field reconstruction method.

#### 2.1.1. Pulse Reflection Method

**Figure 2:**
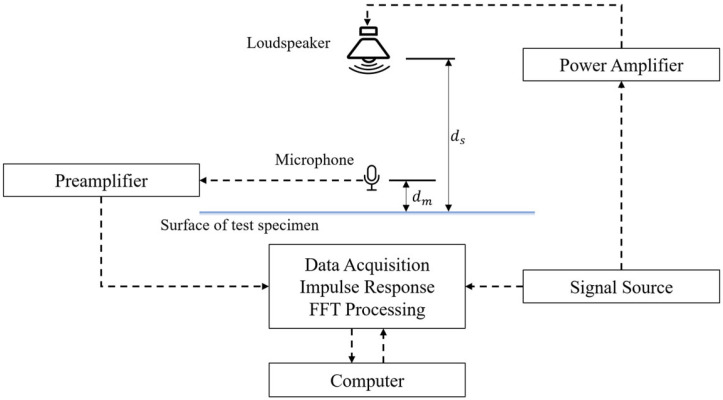
Schematic diagram of the principle of the pulse reflection method system.

The pulse reflection method is an acoustic testing method used for in situ measurement of a material’s sound absorption coefficient, and it is particularly suitable for scenarios where sampling is not possible or testing under laboratory conditions is unfeasible. This method infers the sound absorption performance of the material by analyzing the reflection behavior of sound waves on the material’s surface. The principle is shown in Figure 2. In the measurement principle of the pulse reflection method, the impulse response consists of direct sound, reflected sound, and background noise, which is expressed by the following equation:

(3) $$h_{m}\left(t\right)=h_{i}\left(t\right)+K_{r}h_{i}\left(t\right)\cdot r_{p}\left(t-\Delta \tau \right)+\sum_{j}K_{r,j}h_{i}\left(t\right)\cdot r_{p,j}\left(t-\Delta \tau_{j}\right)+h_{n}\left(t\right)$$

(4) $$K_{r}=\frac{d_{s}-d_{m}}{d_{s}+d_{m}}$$

where $h_{m}\left(t\right)$ is the system impulse response, $h_{i}\left(t\right)$ is the direct sound impulse response, $r_{p}\left(t\right)$ is the specimen reflection coefficient, $h_{n}\left(t\right)$ is the background noise impulse response, $K_{r}$ is the path length difference between the direct pulse and the reflected pulse, $d_{s}$ is the sound distance of the direct pulse (m), $d_{m}$ is the sound distance of the reflected pulse (m), Δτ is the delay generated by the sound from the sound source to the specimen (s), $\Delta \tau =2d_{s}/c$, where c is the speed of sound (m/s), $d_{s}$ is the distance from the specimen to the loudspeaker (m).

Fang et al. [25] used a self-designed duct pulse acoustic experimental system (with a 1 ms Butterworth pulse sound source) to conduct reflection method (closely attached to a rigid backing) and transmission method measurements on 20 mm-thick black polyurethane foam. The experiment showed that the results of the reflection method were consistent with those obtained using the B&K 4206 impedance tube, with an average sound absorption coefficient of 0.57.

Xie Rongji et al. [26] developed a measurement system based on the pulse reflection method. Using 50 mm-thick dense-cavity sponge as the sample, they conducted in situ measurements in an ordinary room. The results showed good agreement with those of the impedance tube method in the frequency range above 400 Hz.

The pulse reflection method is an efficient, flexible acoustic absorption coefficient measurement method suitable for in situ use. Especially when combined with vector microphones and advanced pulse signal processing technologies, it exhibits significant advantages in anti-interference, calibration-free operation, and wide-frequency-band measurement. Although there are model errors in the low-frequency range, its in situ applicability and rapid measurement capability make it an important supplement to laboratory methods.

#### 2.1.2. Two-Microphone Method

**Figure 3:**
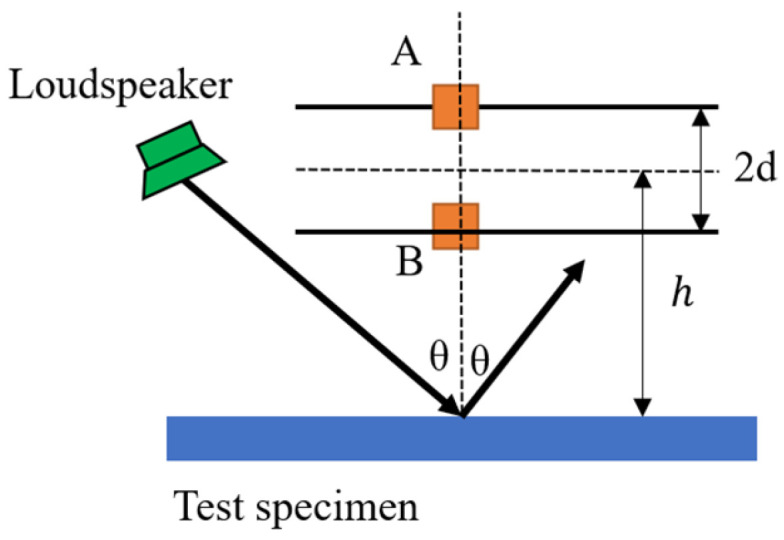
Schematic diagram of the two-microphone method, the vertical dotted lines in the figure represent the plumb direction, and the horizontal dotted lines represent the center line of the distance between the two microphones.

**Figure 4:**
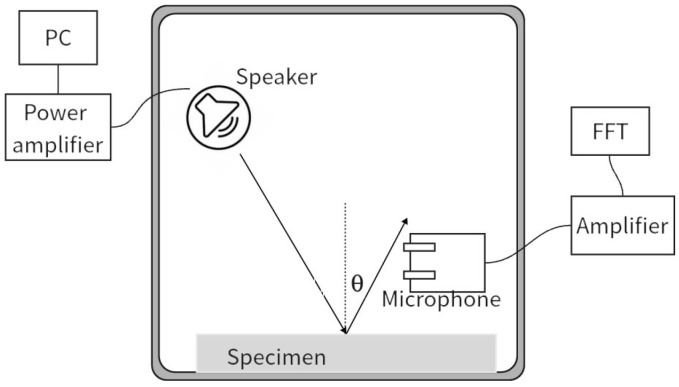
Experimental layout diagram.

The two-microphone method is an acoustic measurement technique used for in situ measurement of the oblique incidence sound absorption coefficient of materials. Chen [27] has provided a detailed description of this method. Its basic principle is to measure the sound pressure signals on the material surface using two microphones, calculate the specific acoustic impedance of the material, and then derive the reflection coefficient and sound absorption coefficient, as shown in Figure 3. Chen’s experimental layout is shown in Figure 4. This method has high accuracy in the low-frequency range and is suitable for rapid in situ detection. Two microphones (A and B) are vertically placed in front of the material surface at a fixed distance to measure the sound pressure signals. The sound absorption coefficient is expressed as follows:

(5) $$\alpha_{a}\left(\theta \right)=1-{\left|\frac{zcos\theta -1}{zcos\theta +1}\right|}^{2}$$

(6) $$z=\frac{1}{\rho_{0}c_{0}}\frac{p}{v_{n}}$$

where $\alpha_{a}\left(\theta \right)$ is the sound absorption coefficient of the material for sound waves at different angles of incidence, θ is the sound wave incidence angle (rad), and z is the specific acoustic impedance (Pa·s/m), $\rho_{0}$ is the density of air (kg/m^3^), $c_{0}$ is the speed of sound in air (m/s), p is the sound pressure (Pa), $v_{n}$ is the normal particle velocity (m/s).

*z* can be measured using the two-microphone method.

The sound pressure $P\left(f\right)$ and particle vibration velocity $V\left(f\right)$ at the midpoint of the connection line between microphones A and B can be calculated:

(7) $$P\left(f\right)=\left[P_{A}\left(f\right)+P_{B}\left(f\right)\right]/2$$

(8) $$V\left(f\right)=\frac{1}{2jw\rho_{0}d}\left[P_{A}\left(f\right)-P_{B}\left(f\right)\right]$$

where $P\left(f\right)$ is the midpoint sound pressure (Pa), $P_{A}\left(f\right)$ and $P_{B}\left(f\right)$ indicate the sound pressure of microphones A and B (Pa), $\rho_{0}$ is the air density (kg/m^3^), d is the distance between microphones A and B (m), w  is the angular frequency (rad/s).

The normalized specific acoustic impedance z can be obtained from $P_{A}\left(f\right)$ and $P_{B}\left(f\right)$:

(9) $$z=\frac{jwd}{c_{0}}\frac{G_{AA}-G_{BB}+j2Im\left(G_{AB}\right)}{G_{AA}+G_{BB}-2Re\left(G_{AB}\right)}$$

where z is the specific acoustic impedance (Pa·s/m), w is the angular frequency (rad/s), d is the distance between microphones A and B (m), $G_{AA}$ and $G_{BB}$ respectively represent the auto-power spectra of $P_{A}\left(f\right)$ and $P_{B}\left(f\right)$, $G_{AB}$ is the cross-power spectrum of $P_{A}\left(f\right)$ and $P_{B}\left(f\right)$, $c_{0}$ is the speed of sound in air (m/s).

Wang [28] conducted a systematic review of various in situ measurement methods, including the two-microphone method. It is pointed out that the two-microphone method was suitable for measuring the oblique incidence sound absorption coefficient, especially performing excellently in the low-frequency range, and was insensitive to the distance between the microphones and the material. This method requires a small sample area, making it suitable for in situ actual measurements, but it needs to be carried out under free-field conditions.

Zhang et al. [29] proposed an in situ sound absorption coefficient measurement method based on sound field reconstruction. The method used a loudspeaker line array combined with the energy ratio-constrained least squares method to reconstruct a plane wave sound field on the material surface, and calculated the sound absorption coefficient using the two-microphone transfer function method. Using melamine foam (50 mm thick) as the sample, experiments showed that when the constraint value was 22 dB, the measurement results in the 160~1600 Hz frequency band were highly consistent with those of the impedance tube method.

Allard [30] studied an improved in situ two-microphone technique for accurately measuring the acoustic surface impedance of materials in a free field. The core of the method is the “spherical decoupling method,” which effectively addresses inaccuracies in traditional plane-wave methods caused by a sound source placed close to the test surface by decoupling sound waves from the source and its image. An iterative procedure further enhances low-frequency accuracy. Experiments demonstrate that this method enables reliable measurements for highly absorbing materials in ordinary rooms, while for weakly absorbing materials, it is feasible at mid-to-high frequencies in large spaces, providing a practical solution for in situ acoustic impedance assessment.

#### 2.1.3. p-u Probe Method

**Figure 5:**
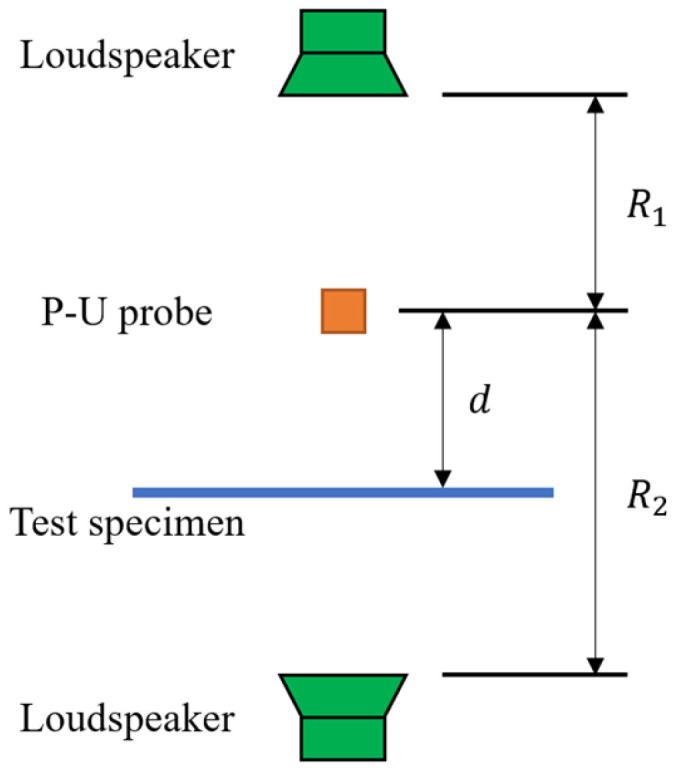
Schematic diagram of the p-u probe method.

A p-u probe is a sensor that can simultaneously measure sound pressure and particle velocity. It can directly calculate sound intensity and sound absorption coefficient, and is equivalent to a simplified version of the two-microphone method. The principle is shown in Figure 5. Duval et al. [31] explored the feasibility of using a p-u probe to measure the sound absorption coefficient in a semi-statistical sound field such as the interior of an automobile. By comparing the “absorbed sound intensity” measured by a large reverberation chamber with that directly measured by the p-u probe, they found that the results of the p-u probe were in good agreement with the simulation results and the reverberation chamber measurement results in the frequency range above 1000 Hz.

Müller-Trapet [32] systematically analyzed the uncertainty factors in in situ acoustic impedance measurements using pu-probes (pressure-velocity probes) through Boundary Element Method (BEM) numerical simulations. The study involved detailed modeling of the geometries of the loudspeaker, probe, and absorber, validated the consistency between simulation and actual measurement results, and focused on investigating the effects of multiple reflections between the probe and the surface, the geometry of the absorber (such as curved surfaces), and the influence of surrounding reflectors on the measurement outcomes.

Lanoye, R. [33] proposed a novel method for measuring the surface impedance and absorption coefficient of sound-absorbing materials in a free field using a combined particle velocity-pressure sensor. The study analyzed the influence of factors such as sensor calibration, source type, source height, angle of incidence, and sample size on the measurement results. The findings indicate that using standing wave tube calibration and the asymptotic solution model yields more reliable results in the low-frequency range. Furthermore, this method enables rapid measurement of surface impedance at different angles of incidence, providing guidance for in situ applications.

Chen et al. [34] presented an in situ technique for measuring sound absorption coefficients using impulse signals and a p-u probe. The method calculated absorption by comparing the surface impedance of the material, derived from simultaneously measured sound pressure and particle velocity, with the free-field impedance using an image-source model. A key advantage is that it avoids the need for sensor calibration and complex separation of direct and reflected sound.

#### 2.1.4. Spatial Fourier Transform Method

**Figure 6:**
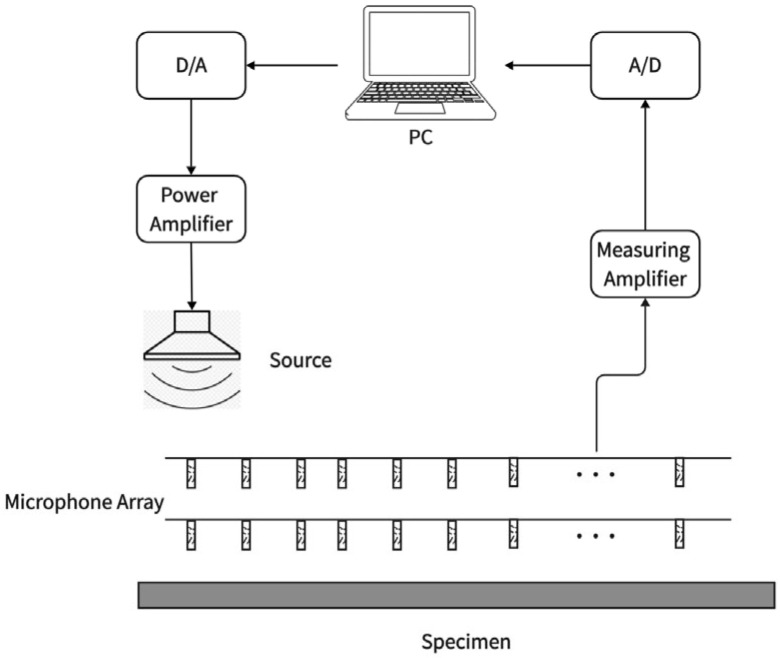
Schematic diagram of the spatial Fourier transform method.

The spatial Fourier transform method is an advanced acoustic measurement technique used to obtain the angle-dependent reflection coefficient of materials, and thereby derive their sound absorption coefficient. The core of this method lies in converting spatially distributed sound pressure signals into the wavenumber domain for analysis, which enables the separation of the incident sound field and the reflected sound field. The laboratory setup for the spatial Fourier transform method is shown in Figure 6. During measurement, a linear microphone array is typically arranged in parallel in front of the material surface to collect complex sound pressure spatial distribution signals on two planes at different heights. Spatial Fourier transforms are, respectively, applied to these two sets of spatial signals, decomposing them into a series of plane wave components with different wavenumbers—each wavenumber component corresponds to a specific sound wave incidence angle. By constructing and solving the simultaneous equations of the wavenumber-domain sound pressure at the two measurement heights, the complex amplitudes of the incident wave and reflected wave corresponding to each incidence angle can be accurately separated. The reflection coefficient can then be directly calculated from the ratio of the complex amplitudes of the reflected wave to the incident wave. A notable advantage of this method is its non-contact and high-efficiency nature: a single measurement can simultaneously obtain the acoustic performance parameters of the material at multiple incidence angles within a wide frequency band. Additionally, it has no special requirements for sound source characteristics, making it highly suitable for studying the angle-dependent properties of non-locally reacting materials [35].

In situ methods offer practical advantages for in situ measurements, but laboratory methods remain the gold standard for accuracy and repeatability, as discussed in the following subsection.

### 2.2. Laboratory Measurement Methods

Laboratory measurements are typically conducted in impedance tubes or reverberation chambers. They provide more accurate and repeatable data under controlled conditions, with high result accuracy.

#### 2.2.1. Impedance Tube Method

##### Standing Wave Ratio Method

**Figure 7:**
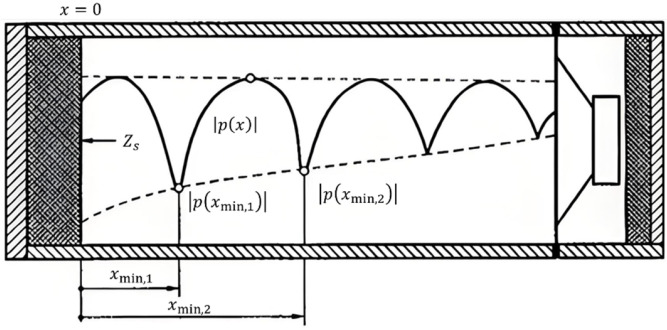
Schematic diagram of the standing wave ratio method.

The principle of the standing wave ratio method is as follows: In a straight, rigid, and airtight impedance tube, a loudspeaker at one end emits a sinusoidal plane wave, as shown on Figure 7. This wave propagates to the surface of the test specimen at the other end and is reflected. The incident wave and the reflected wave superimpose to form a standing wave inside the tube. By measuring the amplitude ratio of the maximum and minimum sound pressure in the standing wave, as well as the position of the first minimum, the acoustic parameters of the material can be calculated [36].

For the incident sound wave:

(10) $$p_{i}\left(x\right)=p_{0}e^{jk_{0}x}$$

where $p_{i}\left(x\right)$ is the incident sound pressure (Pa), $p_{0}$ is the incident sound pressure amplitude (Pa), $k_{0}$ is the wavenumber (rad/s), *x* is the position along tube (m). For a test specimen with a reflection factor of r based on sound pressure, the reflected wave can be expressed as:

(11) $$p_{r}\left(x\right)=rp_{0}e^{-jk_{0}x}$$

where $p_{r}\left(x\right)$ is the reflected sound pressure (Pa), $p_{0}$ is the reflected sound pressure amplitude (Pa), $k_{0}$ is the wavenumber (rad/s), *x* is the position along tube (m), *r* is reflection coefficient.

The specific acoustic impedance of the sound field in a standing wave:

(12) $$Z_{s}\left(x\right)=Z_{0}\frac{p_{i}\left(x\right)+p_{r}\left(x\right)}{p_{i}\left(x\right)-p_{r}\left(x\right)}$$

where $Z_{s}$ is specific acoustic impedance in standing wave (Pa·s/m), $Z_{0}$ is characteristic impedance of air (Pa·s/m), $p_{r}\left(x\right)$ is the reflected sound pressure (Pa), $p_{i}\left(x\right)$ is the incident sound pressure (Pa), *x* is the position along tube (m).

Plane wave sound absorption coefficient:

(13) $$\alpha_{n}=1-{\left|r\right|}^{2}=1-{\left|\frac{{(Z}_{s}/Z_{0})-1}{{(Z}_{s}/Z_{0})+1}\right|}^{2}$$

where $\alpha_{n}$ is the normal sound absorption coefficient, r is reflection coefficient, $Z_{s}$ is specific acoustic impedance in standing wave (Pa·s/m), $Z_{0}$ is characteristic impedance of air (Pa·s/m).

Liu et al. [37] prepared cellular and reticulated titanium foams with high porosity (86~90%) via the improved powder melting and foaming method and the slurry impregnation and sintering method, respectively. He systematically tested the normal sound absorption coefficient of the samples in the frequency range of 200~6300 Hz using the impedance tube method. Cheng et al. [38] measured the normal incidence sound absorption coefficient of three-dimensional reticulated nickel foam by the impedance tube method, with a focus on investigating the sound absorption performance of various multi-layer structures in the frequency range sensitive to the human ear (2000~4000 Hz). Tang [39] used the impedance tube method to measure the sound absorption coefficient of porous materials with different pore structures prepared from 316L stainless steel fibers, and systematically studied the effects of porosity, fiber diameter, material thickness, and the air gap behind the material on sound absorption performance.

Jia [40] tested the sound absorption performance of SAC sponges with different thicknesses in the frequency range of 63~6300 Hz using the impedance tube method. Yu [41] has developed a new type of open-cell metal foam with a windowed structure. Experimental tests using the impedance tube method demonstrated that its average sound absorption coefficient across the entire frequency band increased by 0.2. Using the impedance tube method to measure normal incidence sound absorption coefficients, Liu [42] found that a five-layer stack of 1.5 mm thick, 96% porosity nickel foam achieved an absorption coefficient of about 0.8 at 4000 Hz.

##### Transfer Function Method

**Figure 8:**
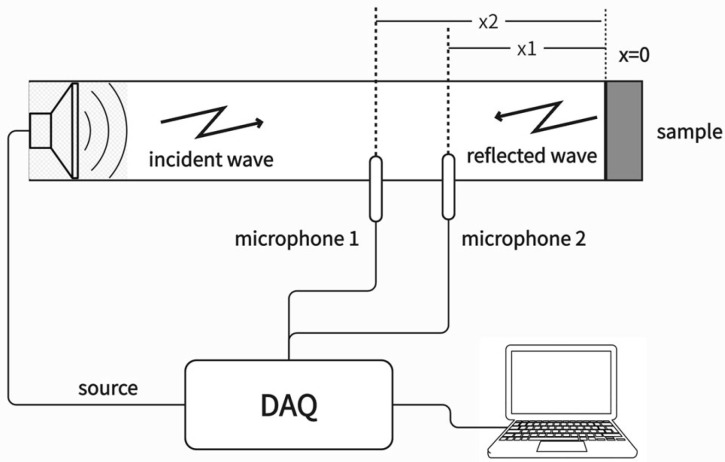
Schematic diagram of transfer function method test.

The transfer function method is a measurement technique that uses two fixed-position microphones (or one movable microphone) in an impedance tube to measure sound pressure signals, as shown in Figure 8. It calculates the complex transfer function via a digital frequency analysis system, and then determines the sound absorption coefficient, specific acoustic impedance, or specific acoustic admittance of a material under normal incidence conditions. During the test, the test sample is installed at one end of the impedance tube, and a sound source generates a plane sound wave. By measuring the sound pressure signals received by the two fixed-position microphones in front of the sample, the complex transfer function between the two signals is calculated. Using this function and sound wave propagation theory, the normal incidence complex reflection factor of the sample surface is derived, and finally the normal incidence sound absorption coefficient and specific acoustic impedance of the material are calculated [43].

Inside the impedance tube, the incident sound pressure $p_{i}$ and the reflected sound pressure $p_{r}$ can be expressed as follows:

(14) $$p_{i}\left(x\right)={\hat{p}}_{i}e^{jk_{0}x}$$

(15) $$p_{r}\left(x\right)={\hat{p}}_{r}e^{-jk_{0}x}$$

where $p_{i}\left(x\right)$ is the incident sound pressure (Pa), ${\hat{p}}_{i}$ is the amplitude of incident sound on the reference plane (Pa), $p_{r}\left(x\right)$ is the reflected sound pressure (Pa), ${\hat{p}}_{r}$ is the amplitude of reflected sound on the reference plane (Pa), and $k_{0}$ is the complex wavenumber (rad/s), x is the position along tube (m).

The sound pressures at the two microphone positions are expressed as:

(16) $$p_{1}={\hat{p}}_{i}e^{jk_{0}x_{1}}+{\hat{p}}_{r}e^{-jk_{0}x_{1}}$$

(17) $$p_{2}={\hat{p}}_{i}e^{jk_{0}x_{2}}+{\hat{p}}_{r}e^{-jk_{0}x_{2}}$$

where $p_{1}$ and $p_{2}$ is the sound pressure of microphone 1 and microphone 2 (Pa), ${\hat{p}}_{i}$ is the amplitude of incident sound on the reference plane (Pa), ${\hat{p}}_{r}$ is the amplitude of reflected sound on the reference plane (Pa), and $k_{0}$ is the complex wavenumber (rad/s), $x_{1}$ and $x_{2}$ is the position along tube of microphone 1 and microphone 2 (m)

The transfer function of the sound wave is:

(18) $$H_{i}=\frac{p_{2i}}{p_{1i}}=e^{-jk_{0}\left(x_{1}-x_{2}\right)}=e^{-jk_{0}s}$$

(19) $$H_{r}=\frac{p_{2r}}{p_{1r}}=e^{jk_{0}\left(x_{1}-x_{2}\right)}=e^{jk_{0}s}$$

where $H_{i}$ and $H_{r}$ are transfer function of the incident sound wave and reflected sound, $p_{1i}$ and $p_{2i}$ are incident sound pressure for microphone 1 and microphone 2 (Pa), $p_{1r}$ and $p_{2r}$ are reflected sound pressure for microphone 1 and microphone 2 (Pa), $s=x_{1}-x_{2}$ is the distance between the two microphones (m). $x_{1}$ and $x_{2}$ are the distances from microphone 1 and microphone 2 to the sample (m), respectively.

The transfer function of the total sound field $H_{12}$ can be expressed as:

(20) $$H_{12}=\frac{p_{2}}{p_{1}}=\frac{e^{jk_{0}x_{2}}+re^{-jk_{0}x_{2}}}{e^{jk_{0}x_{1}}+re^{-jk_{0}x_{1}}}$$

(21) $$\alpha_{n}=1-{\left|r\right|}^{2}=1-{\left|\frac{H_{12}-H_{i}}{H_{r}-H_{12}}e^{2jk_{0}x_{1}}\right|}^{2}$$

where $H_{12}$ is the transfer function of the total sound field, $H_{i}$ and $H_{r}$ are transfer function of the incident sound wave and reflected sound, $p_{1}$ and $p_{2}$ are the sound pressure of microphone 1 and microphone 2 (Pa), $\alpha_{n}$ is the normal sound absorption coefficient, r is reflection coefficient, $k_{0}$ is the complex wavenumber (rad/s), $x_{1}$ and $x_{2}$ is the position along tube of microphone 1 and microphone 2 (m)

Zhang et al. [29] proposed an in situ sound absorption coefficient measurement method based on sound field reconstruction, and calculated the sound absorption coefficient using the two-microphone transfer function method. Gu et al. [44] prepared single-layer and double-layer drainage asphalt rut plate specimens, determined their sound absorption coefficients using the transfer function method, obtained void characteristic parameters by combining (Computed Tomography) CT scanning and 3D reconstruction technology, and established a sound absorption coefficient prediction model based on parameters such as porosity, average volume, and average surface area. Wang et al. [45] used a numerical method to predict the nonlinear sound absorption performance of porous metal layers with finite thickness, and conducted sound absorption coefficient measurements in an impedance tube to verify the numerical results. Ao et al. [46] studied the sound absorption performance of stainless steel fiber porous materials with different thicknesses (1~30 mm), and analyzed the effects of porosity and fiber diameter through impedance tube tests. Huang et al. [47] investigated the sound absorption performance of open-cell aluminum alloy foams with gradient pore sizes. In the experiment, the sound absorption coefficients of the samples were measured in an impedance tube using the transfer function method, and the effects of pore size structure and the thickness of the air gap behind the material on sound absorption performance were systematically analyzed. Li et al. [48] prepared open-cell aluminum foams with spherical pores via the pressure infiltration method, measured the sound absorption coefficients using an impedance tube and the transfer function method, and systematically examined the relationship between their flow resistivity and sound absorption performance. Meng et al. [49] measured the anisotropic acoustic properties of sintered fibrous metals with different thicknesses using the transfer function method in an impedance tube. Xu [50] measured the sound absorption performance of porous materials made from stainless steel fibers with different diameters using the transfer function method in an impedance tube, and investigated the effects of porosity, thickness, fiber diameter, and backing cavity depth on the sound absorption coefficient. Yuvaraj [7] combined open-cell polyurethane foam with closed-cell aluminum metal foam to develop a multi-layer hybrid passive material for automotive noise reduction. The transfer function method of the impedance tube was employed to test the proposed structure and quantify its sound absorption coefficient. Cops [51] measured the normal incidence sound absorption coefficient using an impedance tube (ASTM E1050) for pure metallic foam, pure polyurethane foam, and the composite material. Lin [52] used the two-microphone impedance tube technique to measure the sound absorption coefficient of a sandwich sound-absorbing panel composed of a PET nonwoven layer, a TPU honeycomb grid, and a PU foam layer. The results showed that the sound absorption coefficient of this sandwich panel exceeded 0.93 in the frequency range of 2000~4000 Hz, with a peak value of 0.95 at 3000 Hz, indicating that it had excellent mid-to-high frequency sound absorption performance.

##### The p-u Probe Method in an Impedance Tube

In an impedance tube, a p-u probe (combining a particle velocity sensor and a small sound pressure sensor) is used to simultaneously measure sound pressure and particle velocity. Its principle is similar to that of the transfer function method, with the p-u probe replacing the traditional two microphones. Its accuracy is comparable to that of the two-microphone transfer function method, but it has extremely high requirements for calibration.

(22) $$r=\frac{d-\rho_{0}c_{0}H_{pu}}{d+\rho_{0}c_{0}H_{pu}}e^{j2kd}$$

(23) $$\alpha_{n}=1-{\left|r\right|}^{2}$$

where $\alpha_{n}$ is the normal sound absorption coefficient, r is reflection coefficient, d is the distance from probe to sample (m), $\rho_{0}c_{0}$ is the characteristic impedance of air (Pa·s/m), $H_{pu}$ is the transfer function from the sound pressure to the particle velocity, k is the wave number (rad/s).

Yang Liu and Finn Jacobsen [53] investigated and validated a novel method for measuring the normal-incidence sound absorption coefficient of materials in an impedance tube using a p-u sound intensity probe. This approach simultaneously captured sound pressure and particle velocity at the same position, enabling direct calculation of the reflection coefficient and absorption coefficient without the complex signal processing required in the traditional two-microphone transfer function method. The study demonstrated that, provided the p-u probe was accurately calibrated (amplitude error < 0.5 dB, phase error < 2°), this method could achieve accuracy comparable to the transfer function method. However, the p-u method demanded rigorous calibration and lacked a simple sensor-switching technique to eliminate transducer mismatch, making calibration critical for reliable application.

#### 2.2.2. Reverberation Room Method

The reverberation room method is an international standard method for measuring the random incidence sound absorption coefficient of materials. Its core principle is to calculate the sound absorption performance of a test sample by measuring the change in the room’s reverberation time before and after placing the test sample in a diffuse sound field with uniformly distributed sound energy. Sabine’s formula is used to calculate the equivalent sound absorption area (AT) of the sample. For planar sound absorption materials, the sound absorption coefficient is derived from the following formula:

(24) $$\alpha_{r}=\frac{A_{T}}{S}=\frac{55.3V\left(\frac{1}{c_{2}T_{2}}-\frac{1}{c_{1}T_{1}}\right)-4V\left(m_{2}-m_{1}\right)}{S}$$

where $\alpha_{r}$ is the random incidence sound absorption coefficient, S is the area covered by the sample (m^2^), $A_{T}$ is equivalent absorption area (m^2^), Subscripts 1 and 2 represent the cases of the reverberation chamber without a test piece and the reverberation chamber with a test piece, respectively. V is the volume of the reverberation chamber (m^3^), c is the speed of sound in air (m/s), *T* is the reverberation time (s), m is the sound intensity attenuation coefficient.

The volume of a reverberation room shall be at least 150 m^3^, and its shape shall avoid regular proportions to promote sound field diffusion. Diffusers or rotating vanes shall be used to ensure sufficient diffusion of the sound field. Regarding test samples: For planar sound absorption materials, the area shall be 10~12 m^2^; For discrete sound absorbers (e.g., chairs, screens): They shall be arranged to simulate real-world usage, with a minimum quantity of 3 and a spacing of ≥2 m; The edges of the sample shall be sealed or covered to prevent edge absorption from affecting the results [54].

Two methods are available for measuring reverberation time:

Interrupted noise method: The room is excited with broadband or band-limited noise, and the decay curve is recorded;

Integrated impulse response method: The decay curve is obtained by inverse integrating the squared impulse response, which offers higher accuracy.

The reverberation room method is widely used in the industry. Zhang et al. [55] analyzed and compared the calculation errors generated when using the Sabine formula and the Eyring formula to determine the sound absorption coefficient via the reverberation room method. Through theoretical derivation and numerical comparison, the research results showed that when the average sound absorption coefficient of the material is small, the calculation results of the two formulas are similar; however, when the sound absorption coefficient increases (e.g., >0.2), the Sabine formula produces significant and non-negligible positive errors (the error rate can rise from 5% to over 60%), while using the Eyring formula for calculation can significantly improve the accuracy of the results, making them more consistent with physical reality.

Na. Y et al. [56] systematically investigated the sound absorption properties of micro-fiber fabrics using the reverberation room method. They measured the sound absorption coefficients and noise reduction coefficients of five types of micro-fiber fabrics and one conventional fabric. The results demonstrated that micro-fiber fabrics exhibited superior sound absorption compared to conventional fabrics of the same thickness or weight, and that fabric structure played a crucial role in frequency-dependent sound absorption. The study also identified fabric density as a key factor, with the highest noise reduction coefficient observed at approximately 0.14 g/cm^3^.

E. Toyoda et al. [57] used numerical analysis methods to explore how the shape of the reverberation room and the arrangement of sound diffusion devices inside the room affected the measurement results of the sound absorption coefficient of acoustic materials, and analyzed the relationship between these factors and sound field diffusion. To achieve accurate measurement, irregularly shaped reverberation rooms should be preferred, or diffusers should be properly arranged in regular rooms to enhance sound field diffusion.

### 2.3. Other Measurement Methods

Lou et al. [58] proposed a new method for measuring the normal incidence sound absorption coefficient of non-standard samples whose size was smaller than the cross-section of the impedance tube. The core of this method was to combine the sample with a parallel acoustic material (PAM) of specific acoustic impedance to form a complete test piece. First, the overall acoustic impedance of the test piece was measured, and then the acoustic-electrical analogy method was used to separate and calculate the acoustic impedance and sound absorption coefficient of the non-standard sample itself. The effects of factors such as sample area, shape, boundary length, and PAM characteristics on measurement accuracy were systematically analyzed through numerical simulations and experiments. The results showed that this method could effectively achieve high-precision measurement of non-standard sized samples, especially when a PAM with a similar acoustic impedance was selected.

Sun et al. [59] investigated the sound absorption characteristics of fiber metal materials in high-temperature environments. Based on thermodynamics and variable-property heat transfer theory, they systematically analyzed the mechanism of temperature on acoustic parameters and built a high-temperature impedance tube measurement system for experimental verification. The results indicated that the proposed theoretical model could effectively predict the sound absorption behavior of fibrous materials at high temperatures; the sound absorption coefficient decreased with increasing temperature and was significantly affected by structural parameters such as porosity and fiber diameter.

Yin et al. [60] used the reverberation method to measure the sound absorption coefficient of underwater acoustic materials. The underwater reverberation environment differed greatly from the reverberation environment in air. The physical properties of water resulted in weak diffusion of the underwater sound field, leading to increased uncertainty during measurement. By analyzing different sound source positions, sample areas, and other experimental parameters, an ideal measurement method was proposed, and experimental results within specific frequency bands were presented.

When addressing the systematic errors introduced by improper sample preparation and installation in the measurement of the sound absorption coefficient of underwater materials using the impedance tube method, Sun [61] established a reliable finite element simulation model to systematically analyze and quantify the effects of three key error sources—namely the gap size between the sample and the tube wall, the tilt angle of sample installation, and the surface finish of the sample—on the measurement results. Furthermore, specific parameter constraints for controlling these factors were provided, aiming to effectively reduce systematic errors and significantly improve the reliability and accuracy of underwater sound absorption measurement experiments.

In conclusion, after a long period of evolution and development, the experimental measurement methods for SAC have formed a systematic experimental system. Each experimental method possesses its own unique characteristics and limitations. Engineers need to balance costs and requirements to select the appropriate experimental approach. Various experimental methods mentioned above are summarized and compared herein, as shown in Table 1, with their respective characteristics and limitations highlighted to facilitate readers’ understanding and selection.

## 3. Numerical Calculation Methods for SAC

Numerical methods play a crucial role in the prediction and design of sound-absorbing materials. The following sections discuss various empirical, theoretical, and computational approaches used to estimate the sound absorption coefficient. It is noteworthy that the empirical, theoretical, and computational approaches discussed in this section were primarily developed for, and are most applicable to, dissipative porous materials. The following subsections will detail these methods while explicitly highlighting their intrinsic connection to the acoustic energy dissipation mechanisms in such materials.

### 3.1. Empirical Models

#### 3.1.1. Delany and Bazley Empirical Model

In 1970, Delany and Bazley [13] measured the characteristic impedance and propagation coefficient of a series of commercial fibrous materials through experiments. They found that these parameters could be normalized as a function of the ratio of frequency *f* to flow resistivity σ, and empirical fitting was conducted using a simple power relation. By using only flow resistivity, a key physical parameter, they successfully predicted the acoustic properties of the materials and provided a method to calculate the normal incidence sound absorption coefficient curves of materials with different thicknesses and different flow resistances. Its mathematical model is as follows:

(25) $$Z_{0}=R+jX$$

(26) γ=α+jβ

where $Z_{0}$ and γ denote the specific acoustic impedance (Pa·s/m) and propagation coefficient (m^−1^), j is the imaginary unit, while *R*, X, α and β represent the real and imaginary parts of the specific acoustic impedance and propagation coefficient. Based on the fitting effect, the Delany-Bazley (DB) model can be described as Equations (27) and (28).

(27) $$Z_{0}=\rho_{0}c_{0}\left[1+0.057{\left(\frac{f}{\sigma }\right)}^{-0.754}-0.087{\left(\frac{f}{\sigma }\right)}^{-0.732}j\right]$$

(28) $$\gamma =0.189\frac{\omega }{c_{0}}{\left(\frac{f}{\sigma }\right)}^{-0.595}+j\frac{\omega }{c_{0}}\left[1+0.098{\left(\frac{f}{\sigma }\right)}^{-0.700}\right]$$

where $Z_{0}$ and γ denote the specific acoustic impedance (Pa·s/m) and propagation coefficient (m^−1^), f is the sound frequency (Hz), $\rho_{0}c_{0}$ is the characteristic impedance of air (Pa·s/m), and σ is the flow resistivity (Pa·s/m^2^), ω is the angular frequency (rad/s), j is the imaginary unit.

The DB model was initially established based on experimental data of fibrous materials such as glass fibers and mineral wool, so it was most widely used in the acoustic prediction of fibrous materials. For instance, Bies and Hansen [19] systematically measured the flow resistivity of various commercial fibrous materials, and established an empirical relationship between flow resistivity, density, and fiber diameter based on the DB model. Furthermore, they derived acoustic performance charts for room acoustic design and pipeline linings. When studying functionally graded metal foams, Chua et al. [62] also used the DB model as one of the benchmark models and compared it with the JCA model and neural network models. They found that the DB model still had certain reference value in preliminary design.

#### 3.1.2. Miki Empirical Model

The Miki model [14,15] is a significant modification and extension of the classical DB model, enabling it to be applicable to a wider range of porous materials (including those with different porosities and pore shapes). Moreover, it is suitable for a broader frequency range. The modified model exhibits better performance across the entire frequency band, especially in the range of *f/σ* < 0.01 where the DB model is ineffective. Its mathematical expressions are shown in Equations (29) and (30).

(29) $$Z_{0}=\rho_{0}c_{0}\left[1+0.070{\left(\frac{f}{\sigma }\right)}^{-0.632}-0.107{\left(\frac{f}{\sigma }\right)}^{-0.632}j\right]$$

(30) $$\gamma =0.160\frac{\omega }{c_{0}}{\left(\frac{f}{\sigma }\right)}^{-0.618}+j\frac{\omega }{c_{0}}\left[1+0.109{\left(\frac{f}{\sigma }\right)}^{-0.618}\right]$$

where $Z_{0}$ and γ denote the specific acoustic impedance (Pa·s/m) and propagation coefficient (m^−1^), f is the sound frequency (Hz), $\rho_{0}c_{0}$ is the characteristic impedance of air (Pa·s/m), and σ is the flow resistivity (Pa·s/m^2^), ω is the angular frequency (rad/s), j is the imaginary unit.

**Figure 9:**
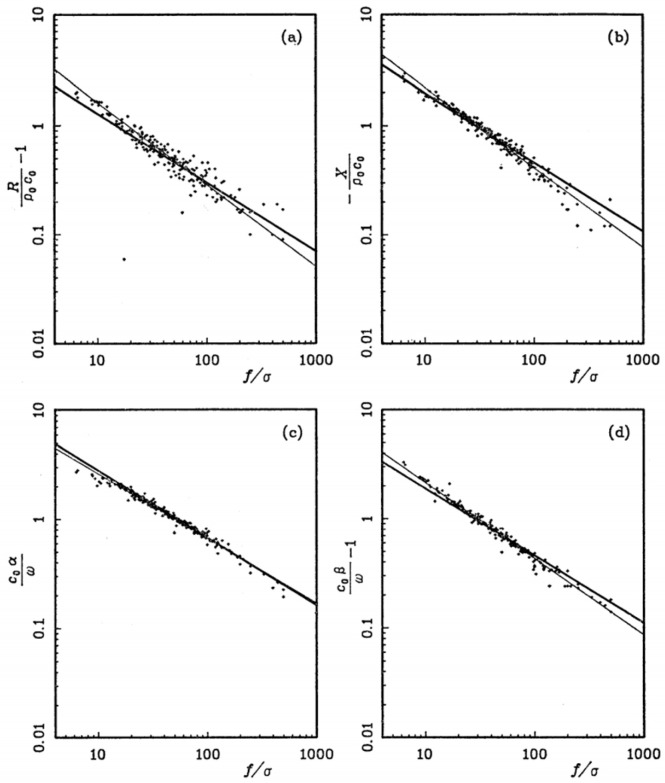
Fitting effects of (**a**) the real part of the specific acoustic impedance, (**b**) the imaginary part of the specific acoustic impedance, (**c**) the real part of the propagation coefficient, (**d**) the imaginary part of the propagation coefficient for the DB and Miki models. The thin lines represent the DB model. Dots show the experimental data by Delany and Bazley. The thick lines represent the Miki model [15].

The fitting effects of the DB method and the Miki method on the real parts and imaginary parts in Equations (25) and (26) are shown in Figure 9.

#### 3.1.3. Komatsu Empirical Model

Komatsu [16] measured the flow resistivity and acoustic properties (characteristic impedance and propagation constant) of 15 types of glass wool and 9 types of rock wool through experiments. It was found that the classical DB and Miki models had relatively large prediction errors in the range of *f/σ* < 0.01 m^3^/kg or *f/σ* > 0.1 m^3^/kg. He proposed a new empirical model including a common logarithmic term, as shown in Equations (31) and (32). The prediction curve of this model is in better agreement with the measured data, which significantly improves the prediction accuracy of acoustic parameters for high-density and low-density fibrous materials in the high-frequency and low-frequency bands.

(31) $$Z_{0}=\rho_{0}c_{0}\left[1+0.00027{\left(2-log\frac{f}{\sigma }\right)}^{6.2}-0.0047{\left(2-log\frac{f}{\sigma }\right)}^{4.1}j\right]$$

(32) $$\gamma =0.0069\frac{\omega }{c_{0}}{\left(2-log\frac{f}{\sigma }\right)}^{4.1}+j\frac{\omega }{c_{0}}\left[1+0.0004{\left(2-log\frac{f}{\sigma }\right)}^{6.2}\right]$$

where $Z_{0}$ and γ denote the specific acoustic impedance (Pa·s/m) and propagation coefficient (m^−1^), f is the sound frequency (Hz), $\rho_{0}c_{0}$ is the characteristic impedance of air (Pa·s/m), and σ is the flow resistivity (Pa·s/m^2^), ω is the angular frequency (rad/s), j is the imaginary unit.

By introducing a logarithmic function, this model has successfully improved the prediction accuracy of the DB and Miki models under extreme *f/σ* values. It is suitable for predicting the acoustic properties of dissipative materials (such as fibrous materials), and performs particularly well in high-density and low-density materials.

While empirical models offer simplicity and efficiency, accurate prediction of acoustic properties also depends on reliable input parameters, such as flow resistivity, which we discuss next.

### 3.2. Theoretical Model

#### 3.2.1. Johnson-Champoux-Allard Theoretical Model

Among theoretical models, the Johnson-Champoux-Allard (JCA) model stands out for its ability to account for both viscous and thermal effects, making it widely applicable across various porous materials. In 1978, Johnson et al. [18] established a rigorous dynamic permeability theory for viscous effects, introducing a characteristic length *Λ* to describe the high-frequency boundary layer behavior and proposing a broadband model that depends solely on geometric parameters. In 1991, Champoux and Allard [17] inherited and extended this framework, applying it to the field of thermal effects. By analogy, they introduced the thermal characteristic length *Λ*^′^, thereby constructing a symmetric model for the dynamic bulk modulus. Together, these developments formed the classical JCA model, as shown in Equations (33) and (34). This thus formed a theoretical framework for describing sound wave propagation in air-saturated porous media. The JCA model describes sound propagation in porous materials by introducing five microstructural parameters (porosity, flow resistivity, tortuosity, viscous characteristic length, and thermal characteristic length). This model treats the material as an equivalent fluid, calculates its equivalent density and equivalent bulk modulus, and then predicts the acoustic impedance, propagation constant, and sound absorption coefficient. Its advantage lies in the ability to simultaneously account for both viscous losses and thermoelastic effects of sound waves in pores. It is applicable to various dissipative materials such as fibers and foams, and exhibits high prediction accuracy especially in the mid-to-high frequency range. The specific calculation formulas of the JCA model are as follows:

(33) $$\rho \left(\omega \right)=\frac{\rho_{0}\alpha_{\infty }}{\phi }\left[1+\frac{\sigma \phi }{j\alpha_{\infty }\rho_{0}\omega }\sqrt{1+j\frac{4\alpha_{\infty }^{2}\eta \rho_{0}\omega }{\sigma^{2}\Lambda^{2}\phi^{2}}}\right]$$

(34) $$K\left(\omega \right)=\frac{\gamma P_{0}/\phi }{\gamma -\left(\gamma -1\right){\left[1+\frac{8\eta }{j{\Lambda^{\prime }}^{2}C_{p}\rho_{0}\omega }\sqrt{1+j\frac{{\Lambda^{\prime }}^{2}C_{p}\rho_{0}\omega }{16\eta }}\right]}^{-1}}$$

where $\rho \left(\omega \right)$ is effective dynamic density (kg/m^3^), $K\left(\omega \right)$ is effective dynamic bulk modulus (Pa), ϕ is the porosity, σ is the static flow resistivity (Pa·s/m^2^), $\alpha_{\infty }$ is the fiber tortuosity, $\rho_{0}$ is the air density (kg/m^3^), Λ is the viscous characteristic length (m), and η is the air viscosity (Pa·s), $P_{0}$ is the air pressure (Pa), ω is the angular frequency (rad/s), γ is the adiabatic index, $\Lambda^{\prime }$ is the thermal characteristic length (m), and $C_{p}$ is the Prandtl number.

The JCA model has been verified to have high prediction accuracy in multiple studies. For example, through impedance tube experiments. Caniato [63] developed and characterized a novel sustainable open-cell foam material, which was made from a sodium alginate bio-matrix incorporated with recycled marine microplastic waste. The sound absorption coefficient of this material was measured using an impedance tube, and inverse parameter identification was conducted via the JCA model. The results demonstrated that the material exhibited excellent sound absorption performance, with the peak sound absorption coefficient exceeding 0.8. Abdi [64] used the JCA model to predict the sound absorption spectrum of the natural fiber composite material made from chrome-tanned leather shavings and coffee silver skin by fitting the tortuosity and two characteristic lengths. Within the frequency range up to 6 kHz, the predictions of the JCA model were in good agreement with the experimental data.

#### 3.2.2. Extension of the Johnson-Champoux-Allard Model

The JCA model describes viscous effects well, but its description of thermal effects still relies on the assumption of cylindrical pores, which is not sufficiently accurate. In the JCA model, the treatment of thermal effects relies on a direct analogy to the formula for viscous tortuosity. While this approach is practical, its theoretical foundation is relatively weak. In 1997, Lafarge et al. [65] fundamentally strengthened this theoretical basis by explicitly defining the dynamic thermal permeability *k*^′^(*ω*), as shown in Equation (35), which achieves perfect symmetry with the dynamic viscous permeability proposed by Johnson et al. in terms of both mathematical form and physical status. This study further demonstrates that as the frequency approaches zero, the dynamic thermal permeability tends to a real constant—the static thermal permeability, as shown in Equation (36). Lafarge et al. established a theoretically rigorous framework for thermal effects in porous media acoustics, which is completely parallel to that for viscous effects. This framework is now the widely adopted (Johnson-Champoux-Allard-Lafarge) JCAL theoretical model.

(35) $$\phi \left\langle \tau \right\rangle =\frac{k^{\prime }\left(\omega \right)}{\kappa }\frac{\partial }{\partial t}\left\langle p\right\rangle$$

(36) $$k_{0}^{\prime }=\frac{1}{\Gamma }$$

where τ is the macroscopic excess temperature in air (K), κ is the coefficient of thermal conduction (W/(m·K)), $k^{\prime }\left(\omega \right)$ is the dynamic thermal permeability (m^2^), ϕ is the porosity, $\frac{\partial }{\partial t}\left\langle p\right\rangle$ is the time rate of change in the macroscopic pressure (Pa/s). $k_{0}^{\prime }$ is the static thermal permeability (m^2^), Γ is the trapping constant of the material (m^−2^).

Based on the equivalent fluid theory, the JCA model treats porous materials as a type of fluid with equivalent density and equivalent bulk modulus, thereby simplifying the modeling of acoustic responses to complex microstructures. However, The JCA model exhibits significant errors in the low-frequency range. In 2007, Kino [66] improved the Johnson-Allard model for rigid-framed fibrous materials. They found that the prediction error of the JCA model exceeded 20% in some cases. Therefore, Correction factors *N*_1_ and *N*_2_, which were dependent on flow resistivity, were introduced by Kino. These factors were obtained through experimental data fitting, as shown in Equations (39) and (40), thereby enhancing the model’s adaptability to materials with different flow resistivities. The high-frequency and low-frequency asymptotic behaviors of effective density and bulk modulus were corrected. The improved model reduced the prediction error of the normal incidence sound absorption coefficient to less than 5% in the frequency range of 800 Hz to 5 kHz, significantly enhancing the prediction accuracy for low-flow-resistivity materials.

The improved model is as follows:

(37) $$\rho \left(\omega \right)=\frac{\rho_{0}\alpha_{\infty }}{\phi }\left\{1+\frac{\sigma \phi }{j\alpha_{\infty }\rho_{0}\omega }{\left[\sqrt{2\eta \rho_{0}\omega }\frac{\alpha_{\infty }\left(1+j\right)}{\sigma \phi \left(\Lambda /N_{1}^{1/2}\right)}\right]}^{1/2}\right\}$$

(38) $$K\left(\omega \right)=\frac{\gamma P_{0}/\phi }{\gamma -\left(\gamma -1\right){\left\{1+\frac{8\eta }{j{\Lambda^{\prime }}^{2}C_{p}\rho_{0}\omega }{\left[\sqrt{2\eta C_{p}\rho_{0}\omega }\frac{1+\gamma }{8\eta {\left(\Lambda^{\prime }/N_{2}^{1/6}\right)}^{3}}\right]}^{1/2}\right\}}^{-1}}$$

(39) $$N_{1}=8.622\exp\left(1.969\times 10^{-6}\sigma \right)-5.54\exp\left(-3.682\times 10^{-5}\sigma \right)$$

(40) $$N_{2}=560.3\exp\left(-5.565\times 10^{-4}\sigma \right)+50.02\exp\left(-5.127\times 10^{-5}\sigma \right)$$

where $N_{1}$ and $N_{2}$ are correction parameters obtained through data fitting, $\rho \left(\omega \right)$ is effective dynamic density (kg/m^3^), $K\left(\omega \right)$ is effective dynamic bulk modulus (Pa), ϕ is the porosity, σ is the static flow resistivity (Pa·s/m^2^), $\alpha_{\infty }$ is the fiber tortuosity, $\rho_{0}$ is the air density (kg/m^3^), Λ is the viscous characteristic length (m), and η is the air viscosity (Pa·s), $P_{0}$ is the air pressure (Pa), ω is the angular frequency (rad/s), γ is the adiabatic index, $\Lambda^{\prime }$ is the thermal characteristic length (m), and $C_{p}$ is the Prandtl number.

In 2019, building on the classical JCA model, Horoshenkov et al. [67,68] made a series of significant theoretical improvements, aiming to address the issue where multiple parameters in the model had abstract physical meanings and were difficult to directly measure. By analyzing the asymptotic behavior of sound propagation in non-uniform cylindrical pores, Horoshenkov found that the viscous characteristic length, thermal characteristic length, viscous permeability, thermal permeability, and tortuosity could be expressed in terms of the material’s median pore size, standard deviation of pore size distribution, and porosity, as shown in Equations (41)–(45). This key finding reduced the number of independent non-acoustic parameters required to describe the acoustic properties of materials from six (in the original JCA model) to only three. Experimental validation demonstrated that the accuracy of this three-parameter model in predicting the acoustic impedance of various materials (such as granular, fibrous, and foam materials) was comparable to that of the traditional six-parameter JCA model.

(41) $$\alpha_{\infty }=e^{4{\left(\sigma_{s}\log2\right)}^{2}}$$

(42) $$\Lambda =\bar{s}e^{-5/2{\left(\sigma_{s}\log2\right)}^{2}}$$

(43) $$\Lambda^{\prime }=\bar{s}e^{-3/2{\left(\sigma_{s}\log2\right)}^{2}}$$

(44) $$\kappa_{0}=\frac{{\bar{s}}^{2}\phi }{8\alpha_{\infty }}e^{-6{\left(\sigma_{s}\log2\right)}^{2}}$$

(45) $$\kappa_{0}^{\prime }=\frac{{\bar{s}}^{2}\phi }{8\alpha_{\infty }}e^{6{\left(\sigma_{s}\log2\right)}^{2}}$$

where $\alpha_{\infty }$ is the tortuosity, ϕ is the porosity, $\bar{s}\;$ is the material’s median pore size (m), $\sigma_{s}$ is the standard deviation in pore size (m), Λ is the viscous characteristic length (m), $\Lambda^{\prime }$ is the thermal characteristic length (m), $\kappa_{0}$ is the viscous permeability (m^2^), $\kappa_{0}^{\prime }$ is the thermal permeability (m^2^). The equation reflects the numerical relationship between the parameters.

D. Oliva and V. Hongisto [22] systematically evaluated seven classical methods for predicting the acoustic impedance of porous materials based on flow resistivity and material thickness, including the DB, Qunli, Miki, Mechel, Komatsu, and Allard-Champoux methods. They compared the theoretical predictions with a large amount of experimental data. The research results showed that the theoretical method proposed by Allard and Champoux had the highest prediction accuracy, and its total prediction error was less than 0.02 in all 1/3 octave bands.

In summary, numerical methods provide powerful tools for predicting SAC of porous materials. Empirical models like DB, Miki, and Komatsu offer simplicity and computational efficiency, making them suitable for rapid engineering estimations, especially in early design stages. For a more comprehensive and physically grounded prediction, the JCA model stands out by incorporating multiple microstructural parameters to account for both viscous and thermal losses, delivering high accuracy across a broad frequency range for various material types. The choice of numerical method ultimately depends on a trade-off between the required accuracy, available material parameters, and computational resources. The different numerical models have their own advantages and applicable scopes when predicting the sound absorption coefficient. For the sake of clarity, Table 2 summarizes the core input parameters, applicable frequency ranges, and key limitations of the main empirical and theoretical models mentioned above. This allows readers to quickly refer to and select an appropriate model based on material properties, parameter acquisition capabilities, and frequency requirements in practical research.

### 3.3. Numerical Simulation Methods

#### 3.3.1. Representative Unit Cell Estimation

Representative unit cell (RUC) is a fundamental technique in computational micromechanics used to predict the macroscopic effective properties of composite materials. Instead of analyzing a large and complex bulk material, the RUC approach simplifies the problem by identifying a small, statistically representative volume element that captures the essential microstructural features of the composite, such as fiber arrangement, volume fraction, and constituent material properties. The primary advantage of the RUC technique is its ability to bridge the micro-scale and macro-scale, enabling cost-effective virtual testing and material design optimization before physical manufacturing.

**Figure 10:**
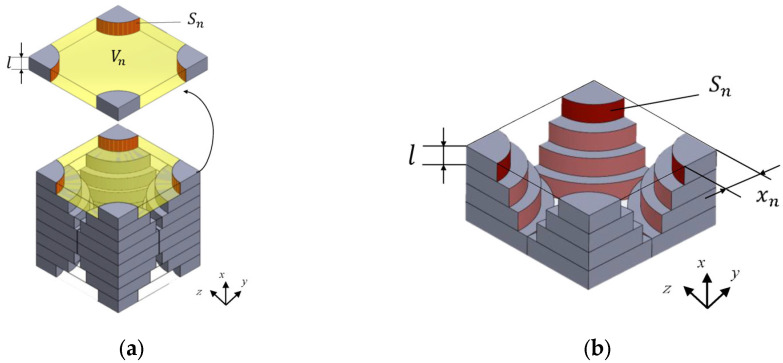
Divided element for the half sphere to (**a**) approximate clearance between the two planes (**b**) cylindrically approximate surface area [69] (reproduced with permission from MDPI).

Sakamoto et al. [69] developed a RUC modeling technique based on micro-CT scan images to accurately predict the sound absorption performance of randomly packed granular materials. This method processes CT tomographic images layer by layer, equates irregular pores to a series of parallel planar slits, and extracts their geometric parameters. The pore cross-sectional area and particle cross-sectional perimeter within each unit are calculated through image processing, as shown in Figure 10. Meanwhile, surface area correction is performed to restore the real three-dimensional structure, achieving accurate and efficient theoretical prediction of the sound absorption coefficient.

**Figure 11:**
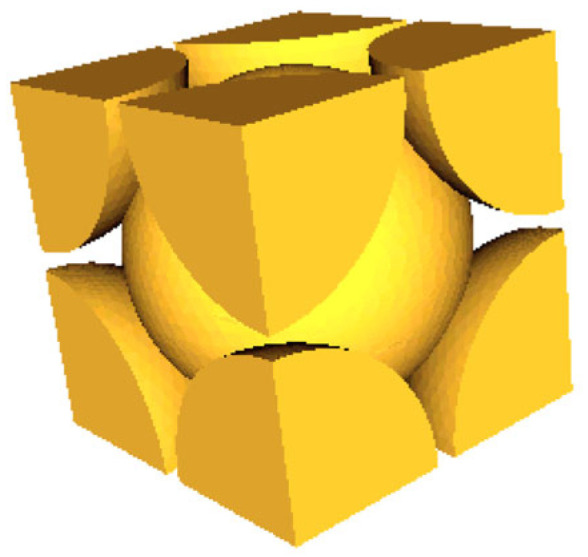
EPS cell for the polystyrene sample with a face-centered cubic lattice [70](reproduced with permission from MDPI).

Meftah [70] successfully characterized the complex pore structure of expanded polystyrene (EPS) foam by combining X-ray micro-computed tomography with saturation technology, and on this basis, applied the RUC technology to predict its acoustic performance. Specifically, the research team clearly distinguished EPS beads from the pore network in micro-CT images. Furthermore, based on the three-dimensional image data, the representative elementary cell of the material was determined, as shown in Figure 11, and the acoustic parameters required for the JCA model (such as porosity, tortuosity, flow resistivity, characteristic length, etc.) were calculated.

For materials with regular structures such as metal foams, an idealized RUC is often used. When studying Inconel 625 foam, Zhai et al. [71] and Yu et al. [72] predicted the flow resistivity based on the geometric parameters of the RUC (such as strut length and width) using the Fourie-Plessis model. They then substituted the predicted flow resistivity into the DB model to calculate the sound absorption coefficient, and the numerical predictions were highly consistent with the experimental measurements.

The flow resistivity is calculated:

(46) $$\sigma =\frac{36\eta \chi (\chi -1)}{\phi^{2}d^{2}}$$

(47) $$\chi =2+2\cos\left[\frac{4\pi }{3}+\frac{1}{3}\cos^{-1}\left(2\phi -1\right)\right]$$

where η is dynamic viscosity of air (N∙s∙m^−2^), χ is a shape factor of the porous structure, d is representative cell size (mm), ϕ is foam porosity.

#### 3.3.2. Computational Fluid Dynamics Simulation Calculation

**Figure 12:**
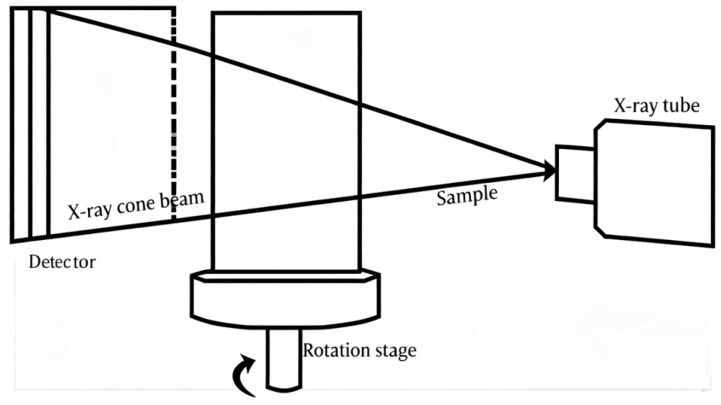
Schematic of the μCT system.

Soltani [73] systematically studied the influence of the microstructural parameters of nonwovens on their sound absorption behavior by combining real three-dimensional microstructural images of nonwovens obtained via X-ray microcomputed tomography (μCT) and computational fluid dynamics (CFD) simulations, as shown in Figure 12. Virtual fibrous structures with different parameters were generated through simulations, and the Stokes equations were solved to calculate the flow resistivity. The study established a 3D reconstructed image of nonwoven fabric. The experimental results were in good agreement with the predictions of the computational model, indicating that the acoustic performance of nonwovens could be effectively optimized by controlling microstructural parameters such as fiber diameter, fabric thickness, and porosity.

The study showed that fabric thickness, fiber diameter, and porosity had significant effects on the sound absorption coefficient. For instance, increasing the fabric thickness could improve the sound absorption effect in the low-frequency range, while reducing the fiber diameter could effectively increase the sound absorption coefficient from the low- to mid-frequency ranges. In addition, the study also found that the directionality of fibers exerted an influence on sound absorption characteristics; particularly, when fibers were oriented along the thickness direction, the sound absorption performance was improved.

Otaru [74] utilized high-resolution X-ray computed tomography (CT) technology to conduct pore-scale structural characterization of Inconel, Recemat, and Porvair foams. Based on these characterization data, key parameters that determined acoustic performance were derived through flow simulation combined with the JCA and DB and Miki models. Experiments showed that the DB and Miki models could well match the normal incidence sound absorption coefficient of glass fibrous materials, while the JCA model was more consistent with the actual test data of metal foams. The predicted sound absorption spectra indicated that the permeability of porous media was the main influencing factor: structures with smaller pore sizes exhibited better sound absorption effects, whereas structures with stronger pore connectivity showed poorer sound absorption effects.

### 3.4. Methods for Calculating Flow Resistivity

Flow resistivity is one of the core non-acoustic parameters that characterize the acoustic performance of dissipative sound-absorbing materials. It describes the material’s ability to resist the passage of airflow and directly determines the intensity of viscous dissipation when sound waves propagate through the pores. Accurately obtaining flow resistivity is crucial for predicting properties of materials such as sound absorption coefficient and acoustic impedance.

#### 3.4.1. Empirical Models for Calculating Flow Resistivity

The empirical method establishes a statistical relationship between flow resistivity and the macroscopic physical parameters of materials (such as density and fiber diameter) based on a large amount of experimental data. Its advantage lies in its simplicity of use, making it suitable for rapid engineering estimation. Through systematic experimental measurements of various commercial fibrous and foams (e.g., glass fibers, rock wool, and polyurethane foam), Bies and Hansen [19] established a classic empirical relationship between flow resistivity, material density, and fiber diameter:

(48) $$\sigma =K_{2}\frac{{\rho_{m}}^{K_{1}}}{d^{2}}$$

where *σ* is the flow resistivity (Pa·s/m^2^), $\rho_{m}$ are the density (kg/m^3^), d is the fiber diameter (m), $K_{1}$ takes values of 1.53 and $K_{2}$ takes values of $3.18\times 10^{-9}$, when using consistent International system of units, for glass fiber materials. The equation reflects the numerical relationship between the parameters.

This formula clearly reveals the physical law that flow resistivity increases with the increase in material density and decreases with the increase in fiber diameter, providing practical charts and a basis for rapid estimation for acoustic engineering design. Materials of different types usually have their specific empirical relationships. For example, when studying kenaf fibers, Ramis et al. [75] initially verified the relationship between flow resistivity, material density, and fiber diameter by combining the Bies-Hansen microstructure model, and proposed special empirical coefficients for this material.

The Bies-Hansen model was developed based on glass fiber materials (with fiber diameters ranging from 1 to 15 μm). It was not applicable to polyester fibers, which were coarser (18–48 μm in diameter), longer, and had fundamentally different flow resistance characteristics from glass fiber materials. Garai and Pompoli [76] proposed a formula for predicting the flow resistance of polyester fiber materials, as shown in Equation (49). This model was fitted based on experimental data from 38 samples, with an average prediction deviation of 9.8%.

(49) $$\sigma =A\rho_{m}^{B}$$

where *σ* is the flow resistivity (Pa·s/m^2^), $\rho_{m}\;$ are the density (kg/m^3^), *A* and *B* are free parameters. Garai and Pompoli found the optimal values of *A* and *B* for polyester fibers were 25.989 and 1.404, when using consistent International system of units. The equation reflects the numerical relationship between the parameters.

Another common classical model is the Kozeny–Carman equation proposed by Kozeny and Carman. Initially, it was used to predict the permeability or flow resistivity of granular media such as soil and sandstone. Later, this model was extended to be applied to fibrous materials. Derived from porous media fluid mechanics, the equation models the material as a bundle of curved capillaries. By comparing different models, Hurrell [77] found that the Kozeny–Carman model was suitable for ideal uniform porous or fibrous media. However, in practical engineering, especially for nonwoven materials with uneven fiber diameters and complex compositions, its prediction accuracy was limited. Thus, it was necessary to combine it with more advanced acoustic inversion models (e.g., the Miki model) to achieve more accurate flow resistivity estimation.

#### 3.4.2. Experiment Method

The International Standard 9053 specifies two methods for the determination of the flow resistivity of porous materials for acoustical applications [78]. The steady-state flow method is designated as the reference method; it operates by establishing and maintaining a constant, steady pressure difference across a test specimen and precisely measuring the resulting volumetric airflow rate through it, with the flow resistivity calculated directly from the ratio of the pressure difference to the velocity. In contrast, the alternating airflow method is classified as an alternative method, which determines the resistance indirectly by measuring the damping of an oscillating air mass in a closed cavity created by the flow through the specimen, a process that is generally faster but requires calibration against the reference steady-state method to ensure accuracy. A widely used standard applied in the industry is ASTM C522-03 [79], whose principle is the same as that of ISO 9053 [78]. Tang [80] made a detailed summary of the commonly used experimental standards for measuring flow resistivity, and the experimental standards frequently applied in the industry are presented in Table 3.

Regarding indirect measurement techniques for airflow resistance, Ingard and Dear [82] proposed a classical method based on a rigidly terminated standing wave tube and the transfer function between two pressure points; Dragonetti et al. [83] developed an alternative approach utilizing the imaginary part of the acoustic transfer function in a two-cavity system. Del Rey’s [84] research found that both the Ingard and Dear method and the Dragonetti et al. method were effective alternatives to complex standardized procedures. They were easier to operate and could provide reliable data in most cases, making them particularly suitable for material screening and routine evaluation. For non-uniform materials, although there was a certain degree of dispersion in the results, these indirect methods could still be used to estimate the possible range of airflow resistance, serving as an effective auxiliary tool for quality control. Furthermore, the acoustic reflection/transmission method infers flow resistivity by solving an inverse scattering problem from reflected or transmitted waves. The comprehensive multi-microphone impedance tube method enables the inversion of flow resistance along with other non-acoustic parameters from directly measured surface impedance and propagation constant [85].

**Figure 13:**
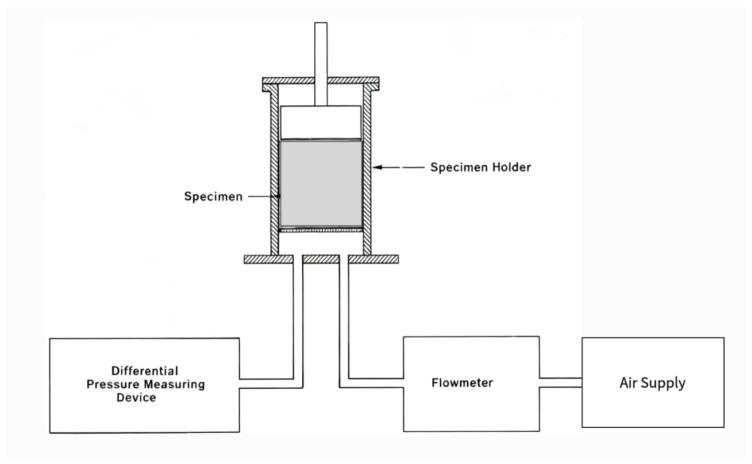
Diagram of experimental apparatus for measuring flow resistivity.

Kino et al. [66] measured the acoustic impedance and physical parameters of 7 types of glass wool and 6 types of polyester fiber samples. They measured the flow resistivity with a device based on the ISO 9053. Lomte [86] adopted the test method specified in the ASTM C522 standard to measure the flow resistivity of foam samples, as shown in Figure 13. The study found that compression led to the deformation of pore structure, a reduction in pore size, an increase in flow resistance, and an enhancement of thermoviscous dissipation. Through compression and a stepped density gradient design, open-cell aluminum foam could significantly enhance its sound absorption and sound insulation performance without a notable increase in weight, providing an effective solution for lightweight noise control in harsh environments such as aerospace.

## 4. Machine Learning Methods for SAC

Building upon the foundation of traditional numerical and experimental methods, recent years have witnessed the rapid emergence of machine learning (ML) as a transformative approach for predicting the sound absorption coefficient. ML techniques can learn complex, nonlinear relationships between material parameters, structural features, and acoustic performance directly from data, often bypassing the need for explicit physical modeling. This data-driven paradigm shows particular promise for optimizing material design and predicting performance where traditional models may be limited.

Unlike traditional empirical or theoretical models that rely on explicit physical laws, ML methods learn complex, nonlinear relationships directly from data. However, the performance and reliability of these ML models are highly dependent on the availability, quantity, and quality of training data. The accuracy of predictions, the model’s ability to generalize to new materials or conditions, and the overall trustworthiness of the results are all intrinsically linked to the dataset used during training and validation. Without sufficient and representative data, even the most advanced ML architectures may fail to capture the underlying acoustic behavior, leading to poor performance or overfitting. Therefore, ensuring access to well-curated, high-fidelity datasets is a critical prerequisite for the successful application of machine learning in acoustic material characterization and design [87].

The following sections explore the application of various machine learning algorithms, including generalized regression neural networks (GRNN), radial basis function networks (RBFNN), artificial neural networks (ANNs), and multiple linear regression, in the realm of acoustic material characterization.

### 4.1. Generalized Neural Regression Network

Zang et al. [88] proposed a method based on the GRNN to predict the sound absorption coefficient of film multi-cavity structural materials. They used 24 sets of film multi-cavity material samples, which included parameters such as different thicknesses, bubble diameters, and porosities, and established two sets of GRNN models. Each set of models was trained using 10 sets of data, and 2 sets of data were used for testing. Experimental measurements showed that the GRNN models could accurately predict the sound absorption coefficients of materials at multiple frequencies, with a maximum error of 0.067, and the prediction curves had a high degree of similarity to the experimental results. This method demonstrated the good performance of GRNN under small-sample data conditions; its fast convergence speed and efficient computing capability made it an effective tool suitable for the rapid evaluation of sound absorption materials.

Mi [89] proposed a hybrid model which integrates the Equilibrium Optimization (EO) algorithm with a GRNN to predict the sound absorption coefficient of three-layer composite aluminum foam. The GRNN was employed for its strong nonlinear mapping capability, while the EO algorithm was used to optimize the key spread parameter in GRNN, significantly improving prediction accuracy especially with small sample sizes. Experimental results demonstrated that EO-GRNN outperformed standalone GRNN and other optimized variants in terms of root mean square error, absolute error, and relative error, achieving an average root mean square error of only 0.011 and exhibiting superior predictive performance.

Liu et al. [90] proposed a general forecasting method based on a GRNN to predict the sound absorption coefficients at six central frequencies (125 Hz to 4000 Hz) and the average sound absorption coefficient of sandwich-structured nonwoven absorbers. The core assumption was that the acoustic properties were determined by easily measured structural parameters—thickness, area density, porosity, and pore size of each layer—given the fiber type. The authors specially designed 100 sandwich absorbers, measured these parameters as GRNN inputs, used the absorption coefficients as outputs, and optimized the GRNN’s spread parameter via cross-validation. Prediction results for 20 test samples demonstrated that the GRNN-based method was reliable and efficient for accurately estimating the acoustic performance of complex structural absorbers.

### 4.2. Radial Basis Function Neural Network

**Figure 14:**
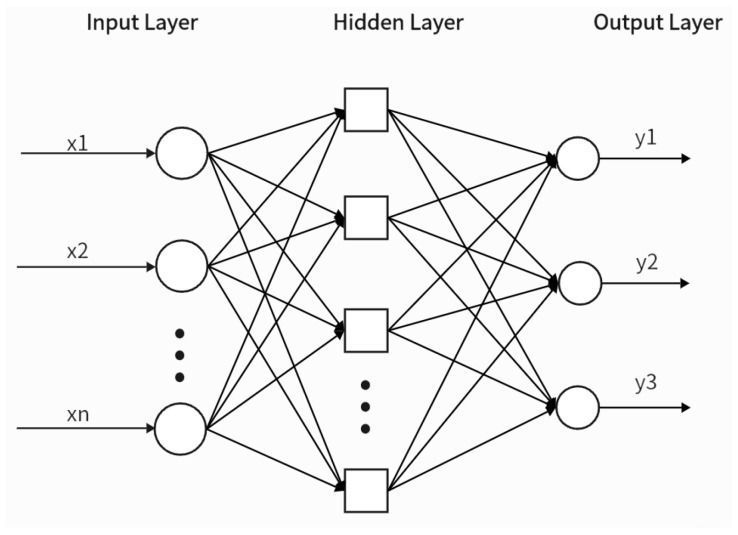
Schematic diagram of radial basis function neural network.

The radial basis function neural network is an effective multi-layer feedforward neural network, featuring optimal approximation capability and excellent nonlinear mapping performance. The basic structure diagram of RBFNN is shown in Figure 14. Composed of an input layer, a hidden layer, and an output layer, the RBFNN has a simple structure and is easy to train. Unlike other neural networks, the hidden layer of the RBFNN adopts radial basis functions (RBFs) as activation functions. These functions only generate significant responses when the input signal is close to the center of the basis function, thereby improving the computational efficiency of the network. The RBFNN is suitable for handling nonlinear problems and has wide applications in complex nonlinear scenarios such as sound absorption coefficient prediction.

Liang et al. [91] investigated the use of RBFNN to predict the sound absorption coefficient of open-cell aluminum foam with composite structures. By designing open-cell aluminum foam composite structures with different thicknesses and densities, they measured structural parameters such as flow resistivity and porosity, and used these data as inputs to train the RBFNN. A comparison was made with the traditional transfer matrix method (TMM) and empirical models (e.g., the DB model, the JCA model). The results showed that the prediction accuracy of the RBFNN in the low-frequency range was significantly better than that of the other methods, with smaller errors. Zhang [92] proposed an optimal design method based on radial basis functions. This method revealed the mechanism by which membrane-type acoustic metamaterials improved the sound insulation dip of double-wall structures, and investigated the newly generated dips as well as the influence of their geometric dimensions.

### 4.3. Artificial Neural Network

**Figure 15:**
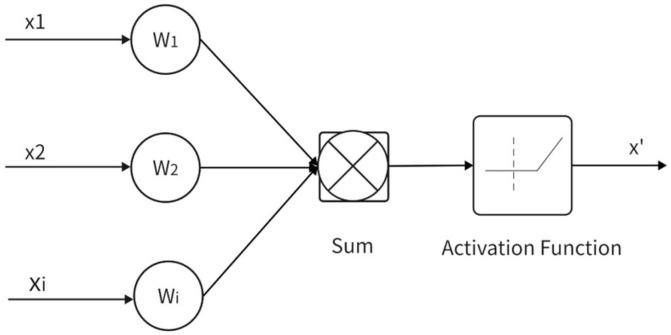
Schematic diagram of neuron unit.

ANNs are computational models that simulate the structure and function of biological neural networks (e.g., the human brain), as shown in Figure 15. They are widely used in fields such as pattern recognition, data classification, and prediction. ANNs consist of nodes arranged in multiple layers, typically including an input layer, a hidden layer, and an output layer. Each node receives input signals, processes them, and the output signals are transmitted through connections between layers, ultimately generating output results. A key characteristic of neural networks is their ability to adjust the connection weights within the network through training, thereby learning and fitting complex patterns in data. The training process usually employs the backpropagation algorithm, which updates the weights by minimizing the output error.

Jeon et al. [93] mainly explored the use of ANNs to estimate the sound absorption coefficient of layered fibrous materials. In the study, the authors trained the neural network model by inputting the material’s structural parameters (such as porosity, thickness, etc.) and other relevant factors to predict the acoustic performance of the material at different frequencies. By comparing with traditional calculation methods, the study found that the ANN model exhibited high accuracy in predicting the sound absorption coefficient, and the neural network demonstrated its superior capability especially when dealing with nonlinear relationships. This method provided a new and effective tool for the rapid evaluation of acoustic material performance.

Ciaburro et al. [94] proposed a new method for predicting sound absorption performance based on ANNs. They used an ANN model to predict the SAC of composite materials, trained the neural network with experimental data, and combined different material properties (such as material porosity, thickness, density, etc.) to establish an ANN model that could effectively predict the acoustic performance of composite materials. The established ANN model had high accuracy and generalization ability, and could effectively predict the sound absorption coefficient of materials in the absence of experimental tests.

Lin et al. [95] used artificial neural networks to estimate the sound absorption coefficient of perforated materials. They collected experimental data of perforated materials with different parameters (such as pore diameter, thickness, material density, etc.), trained an ANN model, and used it to predict the acoustic performance of these materials at different frequencies. By comparing with traditional theoretical models, the study found that the ANN model could predict the sound absorption coefficient of materials more accurately, and showed advantages especially when dealing with complex nonlinear relationships.

Gao et al. [96] proposed a method based on deep neural networks for predicting the low-frequency sound absorption coefficient of underwater coatings. By combining the effective medium theory and the transfer matrix method, the study established an acoustic theoretical model to calculate and solve parameters such as the sound absorption coefficient, surface characteristic impedance, and equivalent bulk longitudinal wave modulus. Using the hypercube sampling method, 20 sensitive parameters were selected, and a database of 100,000 sound absorption coefficient curves (frequency range: 1 Hz~1000 Hz) was constructed. Then, an ANN model was used to predict the average value of the sound absorption coefficient. The verification results showed that the error between the predicted values and the actual values was very small, with a maximum error of 0.33%, which proved the high accuracy and practicality of this method.

Otaru [97] combined numerical simulation with the machine learning backpropagation algorithm to analyze the sound absorption characteristics of honeycomb-type sound insulation materials. ANN model was used to train and analyze the numerical simulation data, and a prediction model based on the material’s non-acoustic parameters was established. The results showed that the ANN model could well predict the sound absorption characteristics of most materials, and the predicted values were close to the actual values, with particularly excellent performance in materials with small pores.

Zhang [98] proposed a method for predicting the sound absorption performance of microporous structures based on an integrated deep neural network (IDNN) model. A database containing 30,744 samples was established to predict the acoustic performance of microporous plates, and training and optimization were conducted by integrating multiple ANN models. Compared with the traditional single ANN model, the IDNN model showed improvements in both the accuracy of frequency prediction and absorption peak prediction: the frequency prediction accuracy was increased by 2.67 times, and the absorption peak prediction accuracy was increased by 3 times. By predicting different data inside and outside the database, the IDNN model demonstrated high prediction accuracy.

Yang [99] proposed a method based on convolutional neural networks (CNNs) for predicting the sound absorption coefficient of metaporous materials. By inputting binary images representing the material’s topological structure into the CNN, the study could effectively predict the sound absorption curves of these materials in the frequency band of 300 Hz to 3000 Hz. The network adopted a deep residual network module, and optimized the model’s hyperparameters through cross-validation of the training and validation sets, ensuring good generalization ability and prediction accuracy.

### 4.4. Multiple Regression

Gu [44] focused on four key void characteristic parameters—calculated porosity, average volume, average surface area, and total void volume—which exhibited a significant correlation with the sound absorption coefficient. Through multiple regression analysis, a multiple linear regression prediction model for the average sound absorption coefficient of drainage asphalt pavement was established. The model expression is as follows:

(50) $$\alpha_{n}=\;-0.651-0.017V+0.003S+1.6\times 10^{-7}V_{t}+0.061\phi^{\prime }$$

where $\alpha_{n}$ is the average sound absorption coefficient, *V* is the average void volume (m^3^), *S* is the average void surface area (m^2^), *V_t_* is the total void volume (m^3^), and *ϕ*^′^ is the calculated porosity.

This model was significant at a 95% confidence level (*p* = 0.008), with all prediction errors below 6%. It exhibited good accuracy and engineering applicability, and could provide a quantitative basis for the noise reduction design of drainage asphalt pavement.

Tang [100] investigated the sound absorption properties of 24 woven fabrics with different structures and developed mathematical models that predicted sound absorption performance based on physical parameters of the fabrics (such as thickness, porosity, air permeability, etc.). The study found that the fabric’s perforation ratio and air permeability were key factors determining sound absorption performance, and reducing these two parameters significantly enhanced the absorption effect. The established models demonstrated high predictive accuracy, with correlation coefficients exceeding 0.77 when validated under different air gap depths of 1 cm, 2 cm, and 3 cm.

Iannace [101] established a generalized linear regression model as a benchmark for comparison. The researchers used the thickness, fiber diameter, flow resistivity, porosity, and frequency as input parameters to predict the sound absorption coefficient of broom fibers. The model was configured with a Gaussian distribution and an identity link function. Evaluation results showed that the linear regression model’s prediction performance was unsatisfactory, with a root mean square error of 0.147, a mean absolute error of 0.130, and a Pearson correlation coefficient of only 0.571. This indicated that the linear model failed to accurately capture the complex relationships between the variables and the sound absorption coefficient.

In summary, ML methods present a paradigm shift from traditional empirical and theoretical approaches for predicting the sound absorption coefficient. Empirical models offer simplicity and computational efficiency but are often limited by their foundational assumptions and narrow applicability to specific material types. Theoretical models provide a more physically grounded framework with higher accuracy across a broader frequency range, yet they require the precise measurement of multiple microstructural parameters, which can be challenging and time-consuming. Experimental methods, such as impedance tube and reverberation room measurements, provide reliable and direct data under controlled conditions, serving as the benchmark for validation; however, they are often time-consuming, costly, and constrained by sample and environmental limitations. In contrast, ML approaches are inherently data-driven. They excel at capturing complex, nonlinear relationships without requiring explicit physical formulations, making them particularly powerful for optimizing multi-layer structures and inverse material design. However, this strength is also their primary constraint: their performance is not governed by physical laws but by the quality and scope of the data they are trained on. While traditional models remain invaluable for providing physical insight and rapid initial estimates, machine learning stands as a complementary and potent tool, capable of uncovering intricate patterns beyond the reach of conventional methods, provided that robust datasets are available to guide its learning [87].

Although ML has shown significant potential in predicting the sound absorption coefficient, it is crucial to recognize their inherent limitations [102]. Firstly, ML models are highly dependent on large quantities of high-quality training data. Insufficient or low-quality data can significantly undermine their prediction accuracy and generalization ability. Secondly, these models are prone to overfitting. In such cases, they may memorize the noise in the training data instead of learning the underlying patterns—this issue is particularly prominent when the available data is limited. Furthermore, ML models are often labeled as “black-box” models, as their internal decision-making processes lack transparency. This opacity impedes the extraction of physical insights related to sound absorption mechanisms. Finally, complex network architectures require substantial computational resources and time for both training and hyperparameter tuning. This can potentially restrict their application in scenarios where resources are constrained.

Therefore, while machine learning serves as a powerful and transformative tool that expands the frontier of acoustic material design, it should be viewed not as a universal replacement for traditional methods, but as a sophisticated complement that is best leveraged when data, computational resources, and a clear understanding of its limitations are in place.

## 5. Comparative Analysis

When selecting a method for predicting or measuring the SAC, researchers and engineers must make trade-offs among accuracy, complexity, cost, time, and practical applicability. This section aims to provide a systematic comparison of the empirical models, theoretical models, experimental measurements, and machine learning methods reviewed in this paper, offering clear guidance for method selection. The core characteristics of these four approaches are summarized in Table 4.

No single method is universally optimal. The choice depends on the specific goals: pursuit of efficiency and simplicity leads to empirical models; the quest for physical insight and high accuracy makes the combination of theoretical models and experimentation the gold standard; confronting massive data and complex nonlinear problems is where machine learning offers a powerful new paradigm. The future trend lies in hybridizing these approaches, for instance, using experimental data to calibrate theoretical models, or incorporating physical constraints into machine learning models, thereby achieving a blend of accuracy, efficiency, and interpretability.

## 6. Conclusions

This review consolidates both traditional and emerging techniques in the field of sound absorption, with an emphasis on the continuous interplay between experimental validations and numerical predictions.

Firstly, experimental methods form the cornerstone of performance evaluation, yet each has a distinct domain of applicability. Laboratory techniques, such as the impedance tube methods, provide unparalleled accuracy and repeatability for normal-incidence SAC under controlled conditions and are indispensable for material development and validation [34,35,36,37,38,39,40,41,42,43,44,45,46,47,48,49,50,51]. In contrast, the reverberation room method offers data for random incidence, which is often closer to real-world applications, but its results are sensitive to room diffusion and sample size [54,55,56,57]. A significant trend is the advancement of in situ measurement techniques. These methods represent a pivotal shift from material characterization in ideal environments to performance evaluation in service conditions, despite challenges related to background noise and sound field complexity [20].

Secondly, numerical methods exhibit an evolutionary path from purely empirical fitting to physics-based modeling. Empirical models like DB, Miki and Komatsu remain valuable for rapid engineering estimation due to their simplicity, but their accuracy diminishes for materials and frequencies outside their original calibration range [13,14,15,16]. The JCA model and its extensions stand out by incorporating microstructural parameters, providing a robust physical framework for predicting SAC across a broad frequency range for various materials [17,22]. A powerful emerging trend is the deep integration of numerical simulation with experimental characterization. Techniques like RUC analysis and CFD enable the prediction of macroscopic acoustic properties directly from a material’s true microstructure, bridging the micro–macro gap and creating new paradigms for material design [69,70,71,72,73,74].

Finally, machine learning has emerged as a transformative, data-driven paradigm. Studies demonstrate that ML models, including GRNN [88,89,90], RBFNN [91], and ANN [93,94,95,96,97,98,99], can capture complex, nonlinear relationships between material parameters, structure, and acoustic performance. They have shown remarkable accuracy in predicting the SAC of complex structures. However, the performance of these ML models is intrinsically linked to the volume and quality of training data, and their “black-box” nature can obscure physical insight [102].

Based on this comprehensive analysis, we identify several promising avenues for future research. The potential for machine learning to optimize sound-absorbing materials by leveraging vast datasets is significant. Future work should focus on integrating the physical constraints of theoretical models (e.g., JCA) into ML architectures. This would create next-generation predictive tools that combine the interpretability of physics-based models with the high accuracy and pattern recognition power of data-driven approaches. In addition, the potential of ML extends beyond performance prediction. Future efforts should leverage generative models and optimization algorithms for inverse design, where a target absorption spectrum is specified, and the model identifies the optimal material microstructure or layered configuration to achieve it.

Furthermore, there is a critical need to quantify the influence of environmental factors on in situ techniques and develop standardized practices. This will facilitate their transition from a research tool to a reliable method for quality control and performance verification in real-world applications. Research should be intensified to develop reliable methods and models for characterizing SAC under challenging conditions such as high temperatures [59], underwater environments [60,61], or high sound pressure levels, catering to the demands of aerospace, marine, and automotive industries.

In conclusion, the field of dissipative sound-absorbing materials is entering an era of methodological convergence. Breakthroughs will increasingly stem from the synergistic combination of controlled experimentation, physics-based numerical modeling, and data-driven machine learning. This integrated approach will enable the more efficient, precise, and targeted development of high-performance acoustic materials for next-generation applications.

## Figures and Tables

**Table 1:** Comparative analysis of experimental methods for SAC.

Method Category	Method Name	Key Advantages	Limitations
In situ methods	Pulse reflection method	suitable for in situ measurement where sampling is impossible; efficient, flexible, and rapid measurement	model errors in the low-frequency range; susceptible to background noise and complex sound fields
	Two-microphone method	suitable for oblique incidence SAC measurement;	must be performed under free-field conditions; limited by background noise
	p-u probe method	directly measures sound pressure and particle velocity; simplified setup compared to two-microphone method	requires high-precision calibration for probe
	Spatial Fourier transform method	non-contact measurement; obtains angle-dependent SAC in a single measurement	requires microphone array and complex processing; laboratory setup may not suit all in situ scenarios
Laboratory methods	Impedance tube (standing wave ratio)	direct and classic method; relatively simple principle and setup	time-consuming point-by-point measurement; requires tube
	Impedance tube (transfer function/two microphones)	fast and efficient broadband measurement; high accuracy and repeatability; international standard	requires tube and calibrated microphones; limited to normal incidence SAC; sample must fit tube cross-section
	Reverberation room method	measures random incidence SAC, closer to real applications; international standard for product rating	requires large, specialized, and expensive room; requires large sample size; sensitive to room diffusion and sample mounting
Other methods	For non-standard samples	enables testing of samples smaller than the tube cross-section or special geometry	accuracy depends on PAM selection and sample geometry
	High-temperature impedance tube	allows for SAC measurement under high temperatures	requires a customized and complex system
	Underwater reverberation method	adapts the reverberation principle for underwater use	weak sound field diffusion increases uncertainty

**Table 2:** Commonly used empirical and theoretical models for SAC.

Model Name	Input Parameters	Validity Range	Limitations
DB empirical model	flow resistivity (*σ*)	0.01 < *f*/*σ* < 1	Inaccurate for extreme *f*/*σ*; limited to fibrous materials
Miki empirical model	flow resistivity (*σ*)	Extended *f*/*σ* range, better at low *f*/*σ*	Still empirical; less accurate for non-fibrous materials
Komatsu empirical model	flow resistivity (*σ*)	Improved for extreme *f*/*σ*	Complexity increases with logarithmic terms
JCA theoretical model	*ϕ*, *σ*, *α∞*, *Λ*, *Λ*^′^	Broad frequency, various materials	Requires 5 microstructural parameters; weak at low frequencies
JCAL theoretical model (Lafarge et al.)	*ϕ*, *σ*, *α∞*, *Λ*, *Λ*^′^, *k*_0_^′^	Improved thermal effects modeling	More parameters; complex calibration
Kino’s modified JCA model	*ϕ*, *σ*, *α∞*, *Λ*, *Λ*^′^, *N*_1_, *N*_2_	800 Hz~5 kHz, low-flow-resistivity materials	Requires correction factors from fitting
Horoshenkov’s modified JCA model	porosity (*ϕ*); median pore size (*x^−^*); standard deviation of pore diameter (*σ_x_*).	Wide range; granular, fibrous, foam materials	Assumes log-normal pore distribution

**Table 3:** Commonly used experimental methods for measuring flow resistivity.

Standard Name	Main Features	Application Scenarios
ISO 9053 Steady-State Flow Method	data direct principle; reference method; technically challenging.	laboratory calibration; material research and development.
ISO 9053 Alternating Flow Method	avoids low-flow measurement; high precision and repeatability; complex equipment.	high-precision measurement; quality control.
ASTM C522-03	equivalent to ISO; industry standard.	industrial testing; North American projects.
ISO 10534-2 Acoustical Transfer Function Method [43]	indirect measurement; model-dependent; inversion calculation.	model parameterization; research and characterization.
ISO 9237 [81]	rapid and simple; derivation from air permeability; approximate estimation.	rapid screening; quality monitoring

**Table 4:** Comparative analysis of different methods for estimating the SAC.

Method Category	Empirical Models (DB, Miki, Komatsu)	Theoretical Models (JCA and Extensions)	Experimental Methods	Machine Learning
Core principle	Statistical regression based on extensive experimental data	Based on the physics of sound propagation in porous media (viscous and thermal effects)	Directly measuring the interaction between sound waves and materials.	Learning complex nonlinear mappings between material parameters and SAC from data.
Typical accuracy	Medium-Low (Significant errors at extreme *f*/*σ* values)	High (Excellent especially in mid-to-high frequencies)	Very high (The standard for validating other methods)	Variable (Data-dependent)
Complexity/Cost	Low (Simple formulas, fast computation)	Medium (Requires multiple microstructural parameters, difficult to obtain; complex model computation)	High (Requires specialized equipment and laboratory environment; time-consuming)	Medium (High upfront cost for data collection and model training; but fast prediction phase)
Key advantages	Requires only flow resistivity (*σ*), extremely simple and efficient; suitable for rapid estimation and preliminary design.	Clear physical meaning, high prediction accuracy, applicable to various materials.	Direct, reliable, most convincing results; standardized methods ensure comparability.	Capable of handling highly nonlinear problems; no need for explicit physical equations.
Key limitations	Applicability limited to the dataset used for model development; accuracy decreases with extreme parameters.	High cost of parameter acquisition; still weak in predicting low-frequency and nonlinear behavior.	Expensive equipment; sample size requirements; in situ measurements susceptible to environmental interference.	“Black-box” nature, lacks physical interpretability; heavily reliant on large volumes of high-quality training data; risk of overfitting.

## Data Availability

Data sharing is not applicable to this article.

## Abbreviations

The following abbreviations are used in this manuscript:

SAC	Sound Absorption Coefficient
DB	Delany-Bazley
JCA	Johnson-Champoux-Allard
JCAL	Johnson-Champoux-Allard-Lafarge
GRNN	Generalized Regression Neural Network
RBFNN	Radial Basis Function Neural Network
ANN	Artificial Neural Network
ML	Machine Learning
RUC	Representative Unit Cell
CFD	Computational Fluid Dynamics
μCT	Micro-Computed Tomography
CT	Computed Tomography
TMM	Transfer Matrix Method
IDNN	Integrated Deep Neural Network
CNN	Convolutional Neural Network
PAM	Parallel Acoustic Material
ASTM	American Society for Testing and Materials
ISO	International Organization for Standardization
